# Ceria–Zirconia-Supported Pt as an Efficient Catalyst for the Sustainable Synthesis of Hydroxylamines and Primary Amines via the Hydrogenation of Oximes Under Ambient Conditions

**DOI:** 10.3390/molecules30091926

**Published:** 2025-04-26

**Authors:** Elena Redina, Inna Ivanova, Olga Tkachenko, Gennady Kapustin, Igor Mishin, Leonid Kustov

**Affiliations:** Laboratory of Development and Study of Polyfunctional Catalysts, N. D. Zelinsky Institute of Organic Chemistry of the Russian Academy of Sciences, 47 Leninsky Prospect, 119991 Moscow, Russia; inigiv022@gmail.com (I.I.); ot@ioc.ac.ru (O.T.); gik@ioc.ac.ru (G.K.); igo@ioc.ac.ru (I.M.)

**Keywords:** heterogeneous hydrogenation, oxime, hydroxylamine, primary amine, platinum

## Abstract

Amines and hydroxylamines are essential compounds in the synthesis of pharmaceuticals and other functionalized molecules. However, the synthesis of primary amines and particularly hydroxylamines remains a challenging task. The most common way to obtain amines and hydroxylamines involves the reduction of substances containing C-N bonds, such as nitro compounds, nitriles, and oximes. Among these, oximes are the most readily accessible substrates easily derived from ketones and aldehydes. However, oximes are much harder to reduce compared to nitro compounds and nitriles. The catalytic heterogeneous hydrogenation of oximes often requires harsh conditions and catalysts with high precious metal loadings, while hydroxylamines are hard to be obtained by this method. In this work, we showed that Pt supported on a porous ceria–zirconia solid solution enables the selective and atom-efficient synthesis of both hydroxylamines and amines through the hydrogenation of oximes, achieving yields of up to 99% under ambient reaction conditions in a “green” THF:H_2_O solvent system. The high activity of the 1% Pt/CeO_2_-ZrO_2_ catalyst (TOF > 500 h^−1^) is due to low-temperature hydrogen activation on Pt nanoparticles with the formation of a hydride, Pt-H. The strong influence of electron-donating and electron-withdrawing groups on the hydrogenation of aromatic oximes implies the nucleophilic attack of hydridic hydrogen from Pt to the electrophilic carbon of protonated oximes.

## 1. Introduction

Amines and hydroxylamines are versatile compounds widely used in both fine and bulk chemical industries. Being intermediate products, amines and hydroxylamines play key roles in the synthesis of crucial substances, including pharmaceuticals [1,2,3,4], metal complex catalysts [5,6,7], polymers [8], and pigments [9,10].

The high reactivity and unique properties of primary amines and hydroxylamines make them valuable classes of compounds [11]. However, the direct synthesis of primary amines and especially hydroxylamines with appropriate yields and purity is still a challenging task. The most effective synthesis strategies are usually based on two-step procedures, aiming at C-N bond formation and its further reduction to form an amino group [12,13]. The reduction of substrates with C-N bonds, such as nitro compounds, oximes, and nitriles, is usually processed with the use of stoichiometric reductants, such as borohydrides [14,15,16] or LiAlH_4_ [17,18,19]. The high toxicity of these reagents and significant waste formation make the overall scheme an inefficient process in light of green chemistry principles. At the same time, the synthesis of nitro compounds and nitriles, especially aliphatic ones, is also not a trivial task, requiring multi-step procedures. The direct reductive amination of carbonyl compounds to primary amines often requires the use of a huge excess of ammonia, and harsh conditions are applied [20,21,22]. In addition, the side reaction of carbonyl compound reduction proceeds.

The catalytic hydrogenation of oximes can be considered as an alternative and more sustainable method for the preparation of primary amines and hydroxylamines with an atom economy of 100%. Oximes are the most synthetically available substrates with a C-N bond that can be easily derived from ketones and aldehydes by condensation with hydroxylamine [23,24,25,26]. Depending on the reaction conditions, further reduction of oximes with H_2_ leads to the formation of corresponding primary amines [27,28,29,30,31], hydroxylamines [32,33], or even piperazines or piperidines through the reductive cyclization of dioximes [3,34]. The simplicity and high selectivity of both steps make this method a convenient pathway, especially in pharmaceutical synthesis, for the production of a variety of primary amines and hydroxylamines, including functionalized, unsaturated, and sterically hindered molecules, as well as N-heterocycles [4,35,36,37].

However, oximes are less reactive compounds compared to nitriles and nitro compounds. As a consequence of charge delocalization due to the resonance effect that decreases the electrophilicity of the carbon atom in the C=N fragment to be attacked by nucleophilic reducing agents, oximes are hard to be reduced [33,38]. High H_2_ pressures and elevated temperatures are usually required for the reaction to proceed [35,39]. An extensive study has been made to discover a suitable catalyst that could promote oxime hydrogenation. A variety of catalysts were reported to possess high activity in oxime reduction, including heterogeneous metal catalysts [35,38,40,41,42], homogeneous catalysts based on transition metal complexes [33,43,44], and organic molecules [45,46].

Chiral cyclometallated iridium-based Cp^X^Ir(III) complexes were found to be efficient catalysts for the enantioselective asymmetric hydrogenation of O-substituted oximes to hydroxylamine derivatives [32]. The reaction proceeds at room temperature with the N-O bond left untouched, yet highly acidic conditions and a 50 bar H_2_ pressure were required. An Ir/Zhaophos catalyst also demonstrated high activity and selectivity in the asymmetric hydrogenation of aromatic ketoximes to chiral hydroxylamines in dioxane with assistance of Lewis In(OTf)_3_ and Brønsted L-camphorsulfonic acids under a hydrogen pressure of 50 atm, and it took 72 h to achieve the full conversion of oximes [33]. A Ni(OAc)_2_·4H_2_O catalyst with an (S,S)-Ph-BPE ligand was also shown to provide the asymmetric hydrogenation of aromatic ketoximes to hydroxylamines, but the reaction should be carried out in a nitrogen-filled glovebox in anhydrous 2,2,2-trifluoroethanol and acetic acid under 50 atm of H_2_ and 50 °C for 24 h [44]. It is evident that homogeneous catalysts are difficult to operate on an industrial scale because of special reaction conditions, poor catalyst recyclability, and product isolation issues [47].

**Scheme 1:**
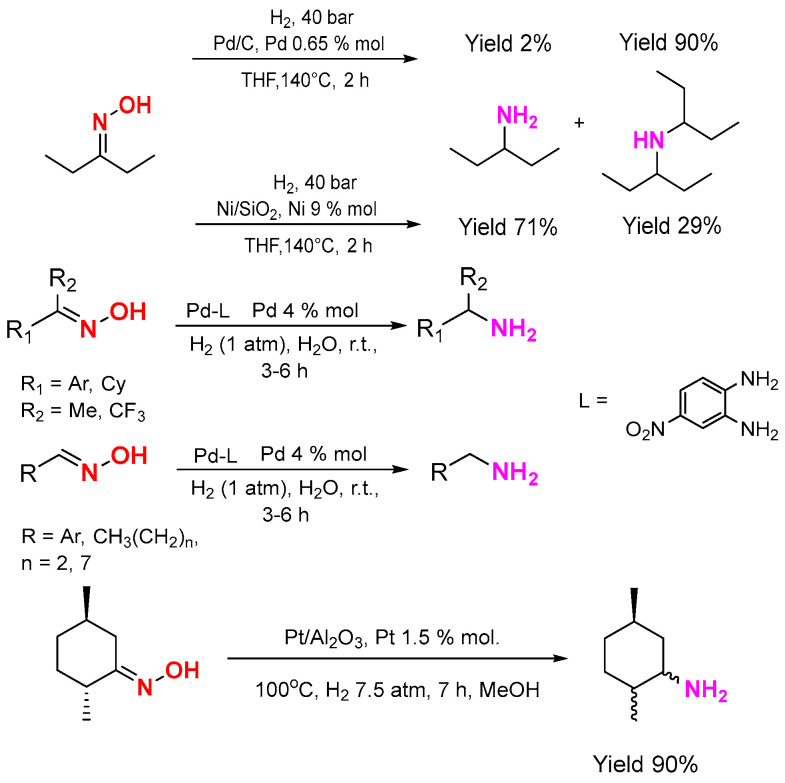
Hydrogenation of oximes over heterogeneous catalysts [29,50,49].

The heterogeneous hydrogenation of oximes is significantly “greener” route to amines or hydroxylamines. Many efforts have been made to perform oxime hydrogenation over heterogeneous catalysts based on transition metal nanoparticles [23,29]. A Ni/SiO_2_ catalyst provides the hydrogenation of 2-ethyl butyraldoxime in 2 h, yet harsh conditions were required (140 °C, 40 atm of H_2_) and a secondary amine was formed together with the primary amine [29]. Notably, Pd/C gave mostly the secondary amine under the same conditions (Scheme 1). Noble metal catalysts can facilitate oxime reduction with H_2_ in acidic media, but the reaction proceeds with poor selectivity. For instance, gold nanoparticles supported on metal oxides (MgO, Al_2_O_3_, ZrO_2_, and TiO_2_) provide the hydrogenation of menthone oxime at 100 °C under a hydrogen pressure of 7.5 bar [48,49]. Apart from the corresponding mentylamine, significant menthone formation was observed, evidencing the side reaction of menthone oxime deoximation. The most promising results were achieved so far in the presence of palladium nanoparticles bound with the 4-nitrobenzene-1,2-diamine ligand. Both aromatic and aliphatic aldoximes and ketoximes were easily hydrogenated to amines in the presence of the Pd/4-nitrobenzene-1,2-diamine catalyst at room temperature and atmospheric pressure [50]. Despite the mild reaction conditions and excellent product yields of up to 99%, the Pd concentration was high (4% mol) (Scheme 1).

It should be specifically noted that there are only a few examples of the heterogeneous hydrogenation of oximes to hydroxylamines, and it required the use of bulk PtO_2_ or 20–100% wt. of Pt/C catalyst [27,51].

The published results on the hydrogenation of oximes over heterogeneous catalysts do not satisfy the principles of green chemistry so far because of the absence of suitable catalytic system characterized by a low amount of precious metal that can selectively operate under mild conditions, resulting in the desired product with high yields.

**Scheme 2:**
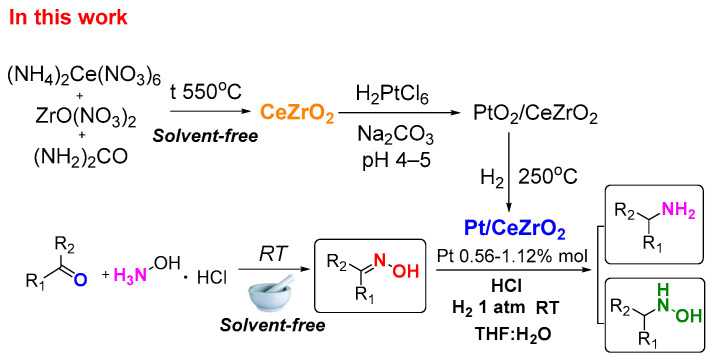
Scheme of this work.

Our previous works have demonstrated that Pt/CeO_2_-ZrO_2_ catalytic system effectively facilitates the selective hydrogenation of carbonyl and nitro compounds to alcohols and amines under ambient conditions [52,53,54]. In this study, we focus on the selective atom-efficient hydrogenation of oximes to hydroxylamines and primary amines under ambient conditions using a recyclable Pt/CeO_2_-ZrO_2_ catalytic system. We emphasize the integration of fundamental organic synthesis with green chemistry principles throughout our approach (Scheme 2). This includes utilizing a green solvent, conducting reactions at room temperature and atmospheric pressure, and employing solvent-free mechanochemical methods for the synthesis of the initial oximes. Moreover, we propose an efficient, waste-free method for synthesizing the CeO_2_-ZrO_2_ support that leads to the development of an active, selective, and stable Pt/CeO_2_-ZrO_2_ catalyst. This holistic approach not only enhances the efficiency of the hydrogenation process but also aligns with sustainable practices in chemical synthesis.

## 2. Results and Discussion

### 2.1. Catalyst Characterization

**Figure 1:**
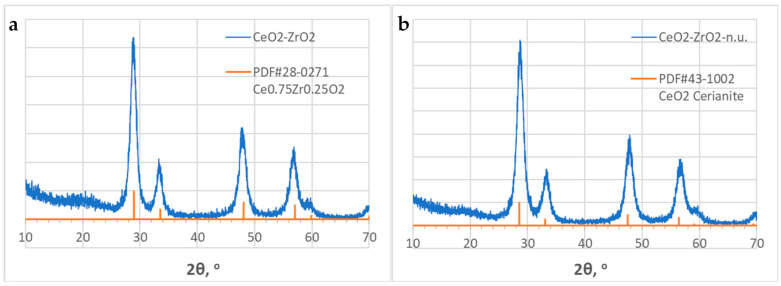
XRD patterns of the CeO_2_-ZrO_2_ (**a**) and CeO_2_-ZrO_2_-n.u. (**b**) samples.

The synthetic procedure for the preparation of the catalysts plays a crucial role in the design of active and selective systems. At the same time, a good synthetic procedure should meet requirements such as simplicity, reproducibility, and low or zero wastes. Therefore, in this work, we have proposed an easy way of CeO_2_-ZrO_2_ support synthesis that implies the convenient calcination of precursor salts in the presence of urea as a pore-forming agent. In our previous works, we have shown that Pt and Cu catalysts supported on CeO_2_-ZrO_2_ mixed oxides with a Ce:Zr ratio of 4:1 w/w showed the best performance in the hydrogenation of carbonyl compounds [52,55,56]. Thus, a CeO_2_-ZrO_2_ support (Ce:Zr = 4:1 *w*/*w*.) was obtained as a solid solution Ce_0.75_Zr_0.25_O_2_ with a coherent scattering region of 10 nm (Figure 1a, Table 1). The method allows the formation of a mesoporous structure of the oxide support with a BET surface area of 70 m^2^/g and pore volume of 0.162 cm^3^/g (Table 2). At the same time, there are only the reflexes of a cerianite phase with a coherent scattering region of 10 nm in the XRD pattern of the CeO_2_-ZrO_2_-n.u. sample prepared without urea (Figure 1b, Table 1); the zirconia phase is amorphous. The pore volume for the sample was almost twice lower (0.096 cm^3^/g) than for the CeO_2_-ZrO_2_ sample prepared with urea, and the specific surface area was only 48 m^2^/g (Table 2).

The coherent scattering region of the pure CeO_2_ sample was a bit larger (14 nm), while the pore volume and specific surface area were found to be lower than for the CeO_2_-ZrO_2_ sample (Table 1 and Table 2).

**Figure 2:**
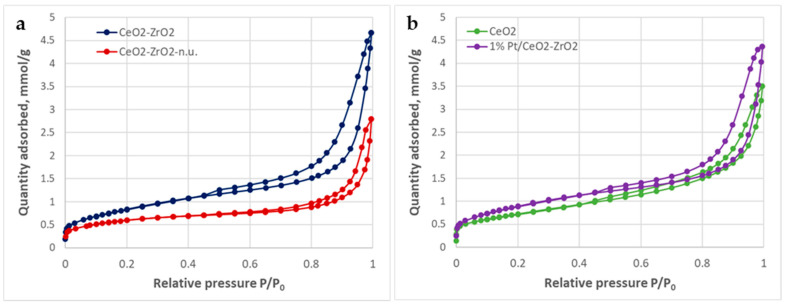
N_2_ adsorption–desorption isotherms for the prepared ceria–zirconia supports (**a**), CeO_2_ (**b**), and 1% Pt/CeO_2_-ZrO_2_ catalyst (**b**).

Previously, it was shown that urea addition greatly improved the porosity of carbon foams obtained by the thermal decomposition of coal tar pitch [57] and polymer membranes for lithium-ion batteries [58,59]. The authors noted that urea thermal decomposition leads to the formation of gaseous products, which leaves the material upon its formation, thus creating a porous structure. The analysis of adsorption–desorption isotherms proves this idea. A type VI isotherm with an H3 hysteresis loop was observed for all prepared samples (Figure 2), which characterized the formation of interparticle pores and the presence of macropores not completely filled with pore condensate [60]. Pt deposition on the obtained CeO_2_-ZrO_2_ oxide did not significantly change the porous structure of the CeO_2_-ZrO_2_ support (Figure 2, Table 2).

**Figure 3:**
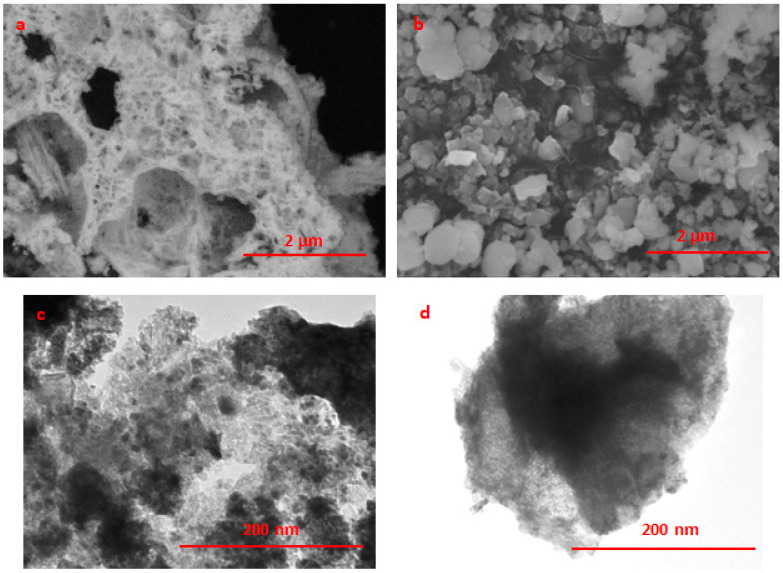
SEM (**a**,**b**) and TEM (**c**,**d**) images of the 1% Pt/CeO_2_-ZrO_2_ (**a**,**c**) and 1% Pt/CeO_2_-ZrO_2_-n.u. (**b**,**d**) samples.

The interparticle pores and support nanoparticles can be seen in the TEM image of the prepared 1% Pt/CeO_2_-ZrO_2_ catalyst (Figure 3c).

Unfortunately, the low Z-contrast between Pt and Ce made it impossible to detect Pt nanoparticles at a such low loading, even by HRTEM [54]. According to the SEM images, the surface morphology of the 1% Pt/CeO_2_-ZrO_2_ catalyst presents a net-like structure with large pores (Figure 3a). The SEM-EDX analysis confirms the uniform element distribution on the catalysts’ surfaces (Figure S2 Supplementary Materials). In contrast, the morphology of the 1% Pt/CeO_2_-ZrO_2_-n.u. catalyst presents a randomly arranged microplates with a smooth surface (Figure 3b). In the TEM image of this sample, interparticle pores can also be recognized, yet of a much smaller size (Figure 3c).

**Figure 4:**
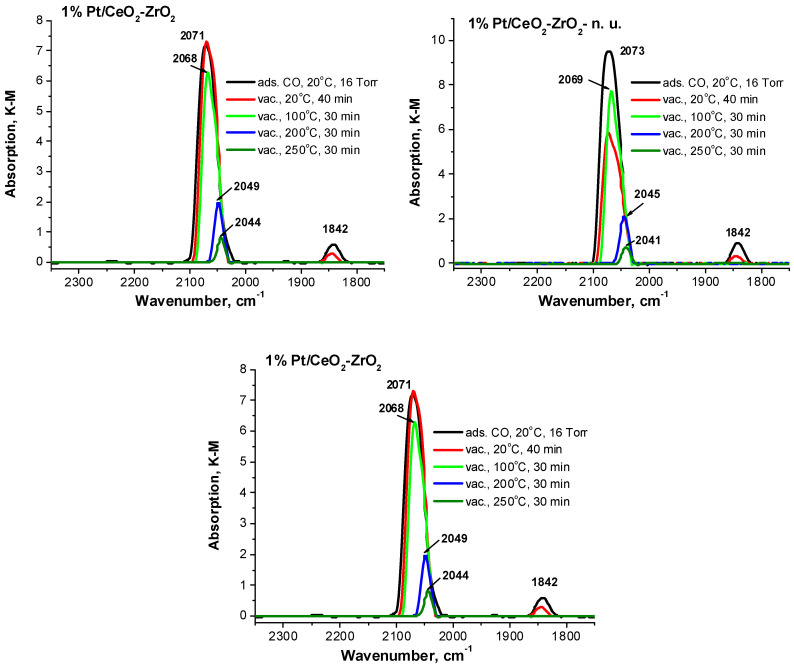
DRIFTS-CO spectra of the prepared catalysts.

The electronic state of Pt supported on the prepared oxides CeO_2_-ZrO_2_, CeO_2_, CeO_2_-ZrO_2_—n.u. was studied by DRIFTS-CO (Figure 4). There are two bands in the spectra of three samples at 2071–2080 cm^−1^ and 1842 cm^−1^. The bands at 2080–2071 cm^−1^ may correspond to CO linearly adsorbed on corner defects of small Pt clusters (less than 55 atoms) with a partial positive charge [54,61,62] and an undercoordinated Pt-O-Pt ensemble [63,64].

The evacuation of CO at temperatures raising from 20 to 250 °C leads to a slow decrease in the intensity of the bands because of the removal of dipole–dipole interactions between the adsorbed CO molecules, and the bands shift to the lower wavenumbers of 2035–2044 cm^−1^, which were previously attributed to CO adsorbed on corners and edges of Pt NPs with a size of 1–1.5 nm [65]. The band at 1842 cm^−1^ is assigned to CO bridged adsorption on Pt NPs.

In DRIFTS-CO spectra of the 1% Pt/ZrO_2_ reference sample, there is also a band at 2044 cm^−1^ and another band at 2190 cm^−1^ (Figure S1 Supplementary Materials) that characterizes the stretching vibrations of the C≡O bond in the carbon monoxide molecule adsorbed on zirconium cations (Zr^4+^-CO) [66].

Therefore, according to the data obtained by DRIFTS-CO, Pt in all samples is present in the form of defect nanoparticles and small clusters with a partial positive charge. The results are in a complete agreement with those we have previously obtained for analogous platinum catalysts on cerium–zirconia oxide supports by DRIFTS-CO and XPS analysis [52,54,67].

**Figure 5:**
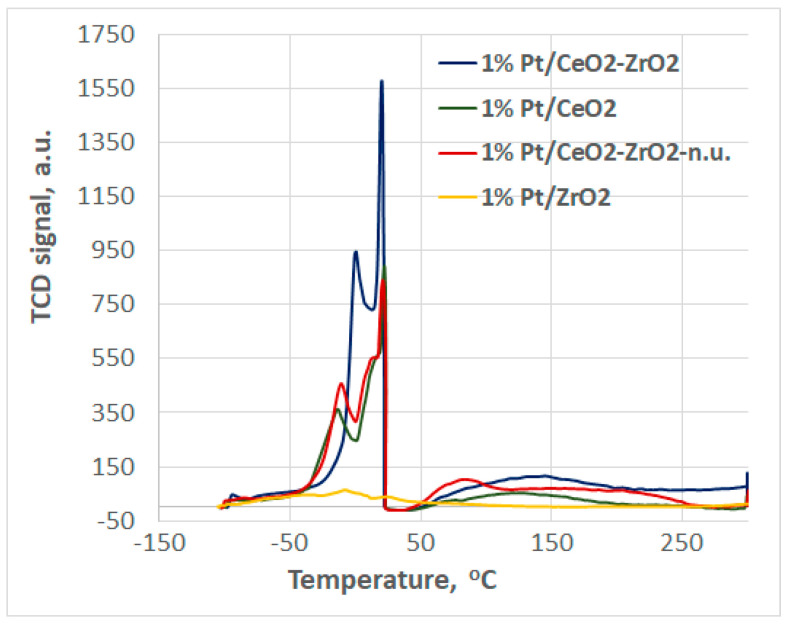
TPR-H_2_ curves obtained for the pre-reduced Pt catalysts supported on different oxides.

The interaction of the catalyst with hydrogen is a key moment in heterogeneous hydrogenation. The obtained catalysts preliminarily reduced in an H_2_ flow at 250 °C were studied in a TPR-H_2_ system in the temperature range of −100–+300 °C (Figure 5, Table 3). We obtained an intensive low-temperature hydrogen consumption at −100–+25 °C for the samples supported on CeO_2_ and CeO_2_-ZrO_2_ oxides; the H_2_ to Pt ratio varied from 5.9 to 8.3 mol/mol (Figure 5, Table 3). This effect is accounted for by the low-temperature giant hydrogen spillover effect we previously detected for Pt catalysts supported on CeO_2_-ZrO_2_ mixed oxides [52,54,62]. The spillover effect is accounted for by dissociative H_2_ chemisorption on Pt and partial CeO_2_ reduction with the hydrogen species formed on Pt NPs that in turn means low-temperature hydrogen activation with the formation of platinum hydride. The formation of Pt-H on Pt supported on the CeO_2_-ZrO_2_ mixed oxide has been proven by SS MAS ^1^H NMR [54]. In contrast, the H_2_/Pt ratio was observed to be 0.2 mol/mol for the 1% Pt/ZrO_2_ and 1% Pt/SiO_2_ samples supported on non-reducible supports.

At the same time, the amount of hydrogen consumed by the samples depends on the way of oxide support preparation. More intensive hydrogen consumption is characteristic of Pt catalysts supported on oxides prepared by precursor salt calcination with urea. Lower specific surface areas and differences in the crystalline structure may be responsible for the lower amounts of defect sites of the support, and thus a smaller perimeter of the Pt-CeO_2_ interface interaction, which leads to less intensive hydrogen spillover [68,69].

### 2.2. Catalytic Results: Hydrogenation of Oximes

The studies of the catalytic activity of the prepared 1% Pt/CeO_2_-ZrO_2_ catalyst, which was characterized by the most intensive low-temperature hydrogen spillover, were started with the reaction of cyclohexanone oxime hydrogenation as an example of aliphatic ketoxime, the reduction of which is usually hindered due to electronic and steric issues. The reaction was first performed in THF under an elevated temperature (60 °C) and H_2_ pressure (20 atm) (Table S1, entry 1, Supplementary Materials), with no substrate conversion having been detected. The addition of proton acid (HCl) significantly enhanced the substrate conversion (up to 35%) (Table S1, entry 2, Supplementary Materials). However, the amine **2b** obtained as a tertiary ammonium salt hardly dissolved in THF; therefore, water was added to THF (1:1, *v*/*v*), which led to the complete disappearance of the characteristic C=N signal (~150 ppm) of the substrate in ^13^C NMR spectrum after 4.5 h of the reaction, and signals at ~50 (CH-NH_2_) and ~53 ppm (CH-NH) appeared, indicating the formation of primary amine **2b** and secondary amine **2c** (Table S1, entry 3). Performing the reaction at RT (25 °C) and H_2_ atmospheric pressure for 4.5 h in the presence of acid also led to complete cyclohexanone oxime conversion with the formation of primary and secondary amines (Figure S5 Supplementary Materials), while no conversion was detected without acid (Table S1, entries 4–5). It is interesting that reducing the time of the reaction to 1 h under ambient conditions made it possible to obtain exclusively N-cyclohexylhydroxylammonium chloride **2a** (Table S1, entry 7, Supplementary Materials) that was isolated in a quantitative yield by simple evaporation of the solvent (after removing the catalyst by centrifugation). Lowering the substrate-to-catalyst ratio allowed the formation of **2b** as the only product both after 4.5 and 2 h, which was further isolated by solvent evaporation in a quantitative yield (Table S1, entries 5–6). Thus, the optimization procedure has revealed the necessity to use a proton acid (HCl) to activate the substrate and the fact that the hydrogenation of the cyclohexanone oxime to amine proceeded consequently through the formation of hydroxylamine as an intermediate product. Activation of the oxime in the presence of a Brønsted acid was previously reported in a number of papers concerning the hydrogenation of oximes [27,32,33]. At the same time, the use of acid makes it possible to obtain amines as salts, which provide their much easier isolation and storage, because amines themselves are hard to store without an inert atmosphere and oxidize, while tertiary ammonium salts can be easily stored for a prolonged time.

Furthermore, we studied the activity of the prepared catalysts in cyclohexanone oxime **1** hydrogenation under the optimal conditions (Table 4). To calculate the TOF values, the reaction was performed for 15 min. Anyway, no conversion was detected in 15 min over the catalysts supported on ZrO_2_ and SiO_2_, for which low-temperature H_2_ spillover was not observed. Only after one hour, the conversion of cyclohexanone oxime **1** and the yield of **2a** appeared to be 20% and 21% on the 1% Pt/ZrO_2_ and 1% Pt/SiO_2_ catalysts with TOF values of 39 and 40 h^−1^, respectively (Table 4, entries 1–2).

For the 1% Pt/CeO_2_ catalysts, the conversion of **1** and the yield of N-cyclohexylhydroxylammonium chloride **2a** in 15 min was 60%, and the TOF reached 428 h^−1^ (Table 4, entry 3). The conversion of **1** and the yield of **2a** over 1% Pt/CeO_2_-ZrO_2_ was 75% in 15 min, with the TOF value being 554 h^−1^ (Table 4, entry 4). After 30 min and 1 h of the reaction, complete cyclohexanone oxime conversion with a quantitative yield of N-cyclohexylhydroxylammonium chloride **2a** was observed over 1% Pt/CeO_2_-ZrO_2_ (Table 4, entries 5–6). At the same time, even after 1 h of the reaction on 1% Pt/CeO_2_, the conversion of **1** was 85%, yet instead of N-cyclohexylhydroxylammonium chloride, amine **2b** was formed (Table 4, entry 7). Performing the reaction on the 1% Pt/CeO_2_-ZrO_2_-n.u. sample led to the complete conversion of cyclohexanone oxime in 1 h, but the catalyst acted not selectively, and a mixture of **2a** and **2b** with another not identified side product was obtained (Table 4, entry 8). No conversion was observed on the commercial hydrogenation catalyst 1% Pd/AC (Table 4, entry 9). Low activity and selectivity were observed for the 1% Pt/C sample (Table 4, entry 10). It took 4.5 h to achieve a conversion of 68%. The yield of **2a** was only 48% and side products were obtained.

Apparently, the composition of the support and its structure influence the catalyst activity and selectivity. The rate of the reaction over the catalysts supported on non-reducible oxides is almost seven times lower, which is in agreement with our previous results on the hydrogenation of carbonyl compounds [52,54]. By means of SS MAS ^1^H NMR, we have found that hydrogen on Pt/CeO_2_-ZrO_2_ was in the form of hydride Pt-H, while on the Pt/SiO_2_ catalyst, hydrogen was detected only in the form of adsorbed molecular H_2_ [54]. This fact explains the higher activity and efficiency of the systems supported on ceria-containing carriers. After all, the hydrogen activation stage is crucial in the hydrogenation reaction. In addition, it is evident that the support takes part in the reaction through the formation of an additional active centers for substrate adsorption and activation.

Performing the hydrogenation of cyclohexanone oxime to N-cyclohexylhydroxylammonium chloride **2a** in a half-gram scale over the 1% Pt/CeO_2_-ZrO_2_ sample gave a 70% yield of the target product in 1 h (Table 4, entry 11).

The stability of the obtained 1% Pt/CeO_2_-ZrO_2_ catalyst was tested in the hydrogenation of cyclohexanone oxime to cyclohexylammonium chloride **2b** (Table 5). The catalyst can be used for at least four times without any noticeable loss in the activity, yet after the 3rd recycle, N-cyclohexylhydroxylammonium chloride **2a** was obtained as the only product under full oxime conversion, indicating that the rate of N-O reduction into hydroxylamine **2a** became slower. An ICP-MS analysis of the fresh and spent 1% Pt/CeO_2_-ZrO_2_ samples revealed a slight decrease in Pt loading in the catalyst from 0.98% to 0.84% wt. (Table S2 Supplementary Materials), which may be responsible for a small loss in the activity during the cycles. The morphology of the catalyst remained unchanged after the repeated cycles (Figure S3 Supplementary Materials), yet Cl was detected on the catalyst’s surface by SEM-EDX (Figure S4 Supplementary Materials). Therefore, washing of the catalyst with water was a necessary step before each cycle.

A number of other ketoximes and aldoximes were hydrogenated over the most active 1% Pt/CeO_2_-ZrO_2_ catalyst. It should be specifically mentioned that some oximes were obtained from the corresponding carbonyl compounds by waste-free and easy-to-handle gram scale mechanochemical synthesis that implies the grinding of a carbonyl compound and hydroxyl amine hydrochloride in an agate mortar (Table S3, pp. S18–S20) [70,71,72].

During the hydrogenation of aliphatic ketoximes (Table 6), an interesting fact was obtained. By varying the substrate-to-catalyst ratio and reaction time, it is possible to obtain a hydroxyl amine or amine as a corresponding hydrochloride salt with good to quantitative yields.

As it was mentioned above, the hydrogenation of cyclohexanone oxime can proceed to N-cyclohexylhydroxylammonium chloride **2a** or to cyclohexylammonium chloride **2b** by simply varying the amount of the substrate from 0.46 to 0.23 mmol and prolonging the reaction time from 1 to 2 h (Table 6, entries 1–2). The hydrogenation of undecan-6-one oxime **3** (0.46 mmol) also proceeds with the formation of hydroxylamine hydrochloride **4**, yet it takes 4.5 h to obtain a complete substrate conversion (Table 6, entry 3). The hydrogenation of 1-phenylbutan-2-one oxime **5** either gives the corresponding N-(1-phenylbut-2-yl)hydroxylammonium chloride **6** in 4.5 h (Table 6, entry 4).

The hydrogenation of aromatic ketoximes drives the formation of primary amines. Thus, the hydrogenation of acetophenone oxime proceeds smoothly and yields the corresponding α-methylbenzylammonium chloride **10** in 2 h (Table 6, entry 6). It should be noted that the presence of an electron-donating group (EDG) in the para-position of ***p***-methyl acetophenone oxime led to a significant decrease in substrate conversion, and the yield of 1-(***p***-tolyl)ethanamine hydrochloride **12** was much lower (Table 6, entry 7). Despite the steric hindrance, the hydrogenation of 1,2-diphenylethanedione monoxime **13** with EWG neighboring the C-N bond yielded 2-hydroxy-1,2-diphenylethylammonium chloride **14**, yet in 6 h with a twice lower substrate-to-catalyst ratio (Table 6, entry 8).

The hydrogenation of cyclododecanone oxime **7** appeared to be slower compared to cyclohexanone oxime because of steric hindrance. Moreover, the selective formation of the hydroxylamine was not observed due to the formation of a corresponding amine, even when the conversion of initial oxime was not complete. Therefore, the primary cyclododecylammonium chloride **8** was isolated in a quantitative yield after 4.5 h (Table 6, entry 5).

The hydrogenation of aromatic aldoximes over 1% Pt/CeO_2_-ZrO_2_ gave the corresponding primary amines (Table 7). The presence of EDG (-OH, -OCH_3_) in the *para*-position of benzaldoxime led to a dramatic decrease in the reaction rate, and the yield of the target amine did not surpass 5% (Table 7, entries 1, 3), while the presence of a -Cl substituent in the *para*-position provided the corresponding ***p***-chlorobenzylammonium chloride **18** in a quantitative yield in 1 h (Table 7, entry 2). Aliphatic aldoximes were successfully hydrogenated to the corresponding primary amines with quantitative yields (Table 7, entries 4–5).

It is important to note that in the case of the hydrogenation of cinnamaldehyde oxime **25** and ***p***-methoxycinnamaldehyde oxime **27**, intermediate hydroxylamines **26a** and **28a,** together with amines **26b** and **28b** were detected in the ^1^H and ^13^C NMR spectra, and the conversion of substrates was >99% in 6 h (Table 7, entries 6–7). The attempts to find the conditions to obtain solely a hydroxylamine product failed because the amine was starting to form when the conversion of the initial oxime was not complete. After all, 3-(4-methoxyphenyl)propylammonium chloride **28b** was obtained as the only product at 40 °C in 4 h with a good yield (Table 7, entry 8).

**Scheme 3:**
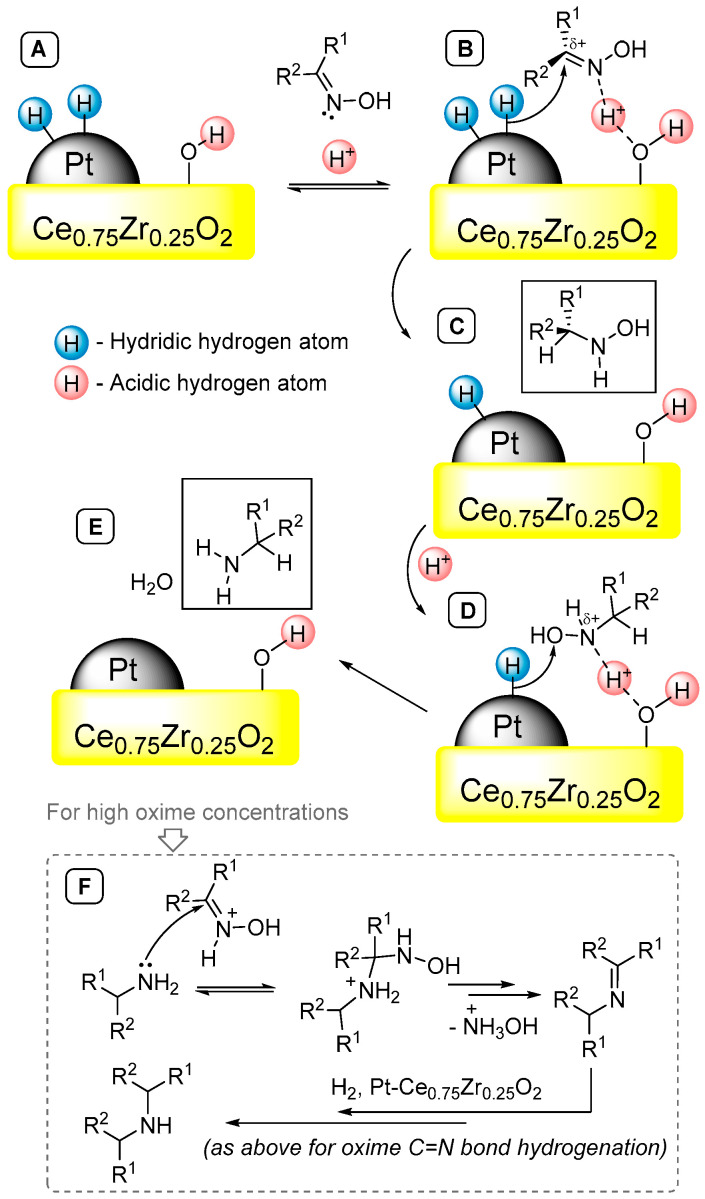
(**A**–**F**)The proposed pathway of the hydrogenation of oximes over the 1% Pt/CeO_2_-ZrO_2_ catalyst.

Based on the experimental results and literature data, the reaction pathway for the hydrogenation of oximes over Pt/CeO_2_-ZrO_2_ was proposed (Scheme 3). The most plausible hydrogenating agents are hydride species formed upon the dissociative adsorption of H_2_ on the Pt surface (**A**). The dissociative character of adsorption and hydride nature of hydrogen on the Pt surface have been shown earlier for the related Pt/CeO_2_-ZrO_2_ system by solid state ^1^H MAS NMR spectroscopy [54]. Moreover, the hydrochloric acid necessary for hydrogenation to proceed under our mild conditions (Table S1 Supplementary Materials) is expected to activate the oxime by N-atom protonation for further nucleophilic attack of hydrogen from the Pt surface (**B**–**C**). Thus, the reaction started with the formation of hydride species on the Pt surface by hydrogen dissociative adsorption and protonation of the N-atom of oxime by hydrochloric acid added to the reaction mixture. The protonated oxime can coordinate to a hydroxy group of the support, as depicted in Scheme 3**B**. It should be noted that both Brönsted and Lewis acid sites can participate in the activation of the oxime toward nucleophilic hydrogen attack; however, only one hydrogen bond type is shown in Scheme 3**B** for simplicity. Then, nucleophilic hydrogen from Pt attacks the carbon with a partial positive charge of the oxime group, which leads to the formation of the hydroxylamine (**C**). Using the example of hydrogenation of aromatic aldoxymes and ketoximes, we have shown that electron-donating substituents (EDGs) in para-position slow down hydrogenation to some extent, while electron-withdrawing groups (EWGs) accelerate the reaction, thus giving a higher conversion and, consequently, yield of the target product at the same reaction time (Table 6, entries 6–7; Table 7, entries 1–3). This is attributed to the contribution of the EWG to withdraw the electron density, thereby increasing the susceptibility for nucleophilic attack on the carbon of C=N-OH group. The observed effects of EDGs and EWGs additionally supports the nucleophilic character of hydrogen addition during hydrogenation of oximes over the Pt/CeO_2_-ZrO_2_ catalyst. The nucleophilic nature of the adsorbed hydrogen on such a system is confirmed by data we obtained for the hydrogenation of carbonyl compounds [52].

Analogously, intermediate hydroxylamine can undergo further hydrogenation to the final amine (**D**, **E**). The formation of the secondary amine at a higher concentration of the oxime and prolonged reaction time (Table S1 Supplementary Materials) can be explained by the acid-catalyzed condensation of the primary amine with the oxime, followed by the hydrogenation of the C=N bond (**F**).

## 3. Materials and Methods

### 3.1. Catalyst Preparation

#### 3.1.1. Support Synthesis

The catalyst support was synthesized by the thermal decomposition of metal salts. To prepare a Ce_0.75_Zr_0.25_O_2_ solid solution (0.940 g), the precursor salts (NH_4_)_2_Ce(NO_3_)_6_ (2.4 g, Acros), ZrO(NO_3_)_2_ (0.272 g, Acros) and urea (10 g, Fluka) were ground in an agate mortar with a pestle. The thermal decomposition was performed in a muffle furnace by heating the mixture to 550 °C (heating rate 1.75 K/min) for 2 h in air. The obtained oxide was denoted as CeO_2_-ZrO_2_. The sample prepared without urea was denoted as CeO_2_-ZrO_2_—n.u. A CeO_2_ sample was obtained by the thermal decomposition of the corresponding precursor salt (NH_4_)_2_Ce(NO_3_)_6_ in the presence of urea. A reference ZrO_2_ sample (92 m^2^/g) was previously obtained by the procedure described elsewhere [52]. The obtained oxides were then used as supports for the catalysts.

#### 3.1.2. Pt Deposition on the Support

To synthesize a 1% Pt/CeO_2_-ZrO_2_ catalyst, 0.88 g of CeO_2_-ZrO_2_ oxide (Ce_0.75_Zr_0.25_O_2_) fine powder was suspended in 88 mL of deionized water (the concentration of the support in the suspension was equal to 10 g/L). Then, 4.55 mL of a 0.0103 M H_2_PtCl_6_ aqueous solution was added to the suspension under continuous stirring at 800 rpm. To initiate the deposition of Pt-containing species on the support surface, the initial solution with a pH = 3.8 was titrated with a 0.1 M aqueous solution of NaHCO_3_ to pH = 5.8. The suspension was then heated up to 60 °C and kept under stirring for 2 h. The process of Pt deposition was monitored by titration of 1 mL of acidified mother liquor with a 0.1 M solution of KI. The complete deposition of PtO*_x_*·H_2_O particles on the CeO_2_-ZrO_2_ support was achieved after 2 h of stirring at pH = 3.95. The suspension was centrifugated to separate the catalyst from the solution. The solid precipitate was washed three times with deionized water and then dried under a vacuum at 40 °C by rotary evaporation. To obtain metallic Pt-nanoparticles, the dried catalyst was reduced in an H_2_ flow (30 mL/min) at 250 °C for 2 h. The same procedure was used to prepare the 1% Pt/CeO_2_ and 1% Pt/CeO_2_-ZrO_2_-n.u. catalysts, and the reference 1% Pt/ZrO_2_ sample.

The reference sample 1% Pt/SiO_2_ was prepared by the wet impregnation of SiO_2_ (KSKG, Russia, S BET = 100 m^2^/g, pore volume BJH 0.664 cm^3^/g) with an H_2_PtCl_6_ aqueous solution (0.0103 M) for 1 h, with subsequent sample drying under a vacuum at 40 °C using a rotary evaporator and further calcination at 250 °C for 2 h. Then, Pt was reduced in an H_2_ flow (30 mL/min) at 250 °C for 2 h.

### 3.2. XRD

The phase composition of the materials and the particle size of the supports were estimated by X-ray diffraction (XRD) analysis. X-ray diffraction patterns were recorded using an ARL X’TRA diffractometer (Thermo Fisher Scientific, Waltham, MA, USA) with Cu Kα radiation (40 kV, 40 mA, λ = 1.54056 Å) and a scanning rate of 1.2° per minute over the range of 10 < 2θ < 70°. Identification of the phases was performed by a comparison of the position and intensity of the peaks with ICSD data. The crystal size of the support nanoparticles was calculated from X-ray peak broadening (Scherer’s equation).

### 3.3. N_2_ Adsorption−Desorption

N_2_ adsorption−desorption isotherms at 77 K were determined by an ASAP 2020 Plus Micromeritics system. Before measuring the adsorption, the calcined sample (0.2−0.3 g) was degassed for 3 h at 300 °C at a residual pressure of 0.8 Pa. The Brunauer−Emmett−Teller method (BET) was used to calculate the specific surface area of the sample.

The pore size distribution for mesopores was determined by the Barrett–Joyner–Halenda (BJH) method applied to desorption isotherms with the Harkins and Jura thickness curve. The total pore volume was estimated at *p*/*p*° = 0.99. The cumulative volume during desorption in the BJH method was taken as the mesopore volume. The micropore volume was calculated by the DFT method (model: N_2_–cylindrical pores–oxide surface). The specific surface area of mesopores was calculated as the cumulative one during desorption in the BJH method. The micropore size distribution was calculated according to the DFT method.

### 3.4. TPR-H_2_

TPR measurements of pre-reduced catalysts were performed in a lab-constructed flow system equipped with a water trap cooled down to –100 °C. The detector (TCD) was calibrated by the reduction of CuO (Aldrich-Chemie GmbH, 99%) pretreated in an Ar flow with a gas flow rate of 30 mL min^−1^ at 300 °C. The Pt/CeO_2_-ZrO_2_ catalyst with a weight of 140–170 mg was pretreated in an argon flow with an oxygen trap at 250 °C for 90 min. Prior to the TPR experiment, the sample was cooled in an Ar flow to—100 °C using a mixture of ethanol and liquid nitrogen as a cooling agent. Heating from –100 to 300 °C was carried out at a rate of 10 °C/min in a 4.6% H_2_–Ar gas mixture purified with an oxygen trap and supplied at a flow rate of 30 mL min^−1^. The sample was kept at room temperature (25 °C) until hydrogen consumption ceased; then, the temperature was raised to 300 °C by applying the same parameters. The obtained data were normalized to 1 g of the sample.

### 3.5. SEM, EDX-SEM, and TEM

A target-oriented approach was utilized for the optimization of the analytical measurements [73].

Before the SEM measurements, the samples were mounted on a 25 mm aluminum specimen stub, fixed by a conductive carbon tape, and coated with a 20 nm film of carbon. The observations were carried out using a Hitachi Regulus 8230 field-emission scanning electron microscope (FE-SEM). Images were acquired in the backscattered electron mode (compositional contrast) at a 20 kV accelerating voltage and in secondary electron at a 3 kV accelerating voltage. EDS-SEM studies were carried out using a Bruker Quantax 400 EDS system equipped with a XFlash 6|60 detector at a 20 kV accelerating voltage.

Before the TEM measurements, the samples were deposited on 3 mm carbon-coated copper grids from an isopropanol suspension. The morphology of the samples wasstudied using a Hitachi HT7700 transmission electron microscope. Images were acquired in the bright-field TEM mode at a 100 kV accelerating voltage.

### 3.6. DRIFTS-CO

Diffuse-reflectance IR spectra (DRIFTS) were recorded at room temperature using a NICOLET Protégé 460 spectrometer in the range of 6000–400 cm^−1^ with a resolution of 4 cm^−1^. For a satisfactory signal-to-noise ratio, 500 spectra were accumulated. The background in the DRIFTS geometry was measured relative to CaF_2_ powder dehydrated in a vacuum and sealed in an ampoule. Carbon monoxide was used as a probe molecule for revealing the electronic state of Pt. Before measuring the spectra, the samples were subjected to vacuum treatment at a temperature of 250 °C for 2 h. The adsorption of CO was carried out at room temperature at an equilibrium pressure of 15 Torr. The band intensities in the spectra were expressed in Kubelka–Munk units. Data collection and processing were performed using the OMNIC program. The spectra of adsorbed CO were presented as the difference between those recorded after and before the adsorption of the probe molecule.

### 3.7. Catalytic Reaction

The liquid phase hydrogenation of oximes was carried out in a 50 mL glass two-necked flask equipped with a three-way glass valve. For a typical experiment, 50 mg of a catalyst was placed in the flask and pretreated in an H_2_ flow for 30 min. The substrate (0.23–0.46 mmol) dissolved in 2–4 mL of a THF and H_2_O mixture (volume ratio 1:1) and a 4.57 M HCl aqueous solution (to obtain an equimolar amount of HCl to the substrate) were then added through the sample valve. The reaction was run at room temperature in an H_2_ atmosphere for 1–6 h under vigorous stirring at 1000 rpm. After the completion of the reaction, the liquid phase was separated from the catalyst by centrifugation and then dried by rotary evaporation. The reaction products were analyzed by ^1^H and ^13^C NMR spectroscopy.

For recycling experiments, the used catalyst was washed with acetone (2 mL) and water (2 mL) three times. After that, the catalyst was dried in an oven at 60 °C for 4 h and then at 110 °C overnight.

### 3.8. NMR and HRMS

^1^H and ^13^C NMR spectra were recorded with a Bruker Fourier 300HD (300.1 and 75.5 MHz) spectrometer in CDCl_3_, DMSO-*d*_6_, and D_2_O. High-resolution mass spectra were recorded on a Bruker MicroTOF II instrument using electrospray ionization (ESI).

In the case of the yield calculation with the use of standard C_2_H_2_Cl_4_, Equations (1) and (2) were applied:

(1) $$n\;prod=n\;st\cdot \frac{I\;prod}{Hprod}\cdot \frac{H\;st}{Ist}$$

*n*—amount of the standard (st) or product (prod) in the probe, mol;

*I*—the intensity of a characteristic signal in the ^1^H NMR spectrum;

*H*—the number of protons correlated to the characteristic signal in the ^1^H NMR spectrum;

(2) $$Y=\frac{n\;prod}{n\;t}\cdot \frac{m\;}{m\;prod}\cdot 100\%$$

*Y*—yield, %

*n t*—the theoretical amount of the target product, moles;

*m prod*—the mass of the probe taken to the NMR analysis, g;

*m*—the overall mass of the dried sample obtained in the reaction, g.

## 4. Conclusions

In this work, we have demonstrated the atom-efficient hydrogenation of oximes in a green H_2_O:THF solvent over 1% Pt/CeO_2_-ZrO_2_ under ambient conditions. The CeO_2_-ZrO_2_ support was prepared by a convenient and waste-free method based on the thermolysis of ceria and zirconia precursors in the presence of urea. This approach allowed the preparation of porous ceria–zirconia oxide with a solid solution structure, the use of which as a support for Pt nanoparticles gave rise to a more effective system compared to Pt on CeO_2_-ZrO_2_ prepared without urea and a Pt/CeO_2_ sample, due to the higher specific surface area and defect support structure because of ZrO_2_ incorporation in the CeO_2_ lattice. Moreover, the activity of the 1% Pt/CeO_2_-ZrO_2_ catalyst was almost fourteen times higher than that of Pt supported on non-reducible SiO_2_ and ZrO_2_, while the conventional hydrogenation catalyst Pd/AC was not active under the conditions used. The reason for the high activity of the 1% Pt/CeO_2_-ZrO_2_ catalyst is accounted for low-temperature hydrogen activation and low-temperature H_2_ spillover. The catalyst can be used for at least four times without a significant loss of activity.

A very important result of the present study is that oxime hydrogenation on 1% Pt/CeO_2_-ZrO_2_ catalysts proceeds via hydroxylamine formation, and it is possible to isolate the hydroxylamine product as a hydrochloride salt with yields of 70–91%. Further hydrogenation of hydroxylamines leads to amines that are obtained as hydrochloride salts with yields up to 99%. Another interesting fact is a strong influence of electronic effects on the hydrogenation of aromatic oximes, indicating the hydride character of hydrogen on Pt NPs, which was proven to be formed on Pt supported on ceria–zirconia oxide [54]. According to the obtained experimental results, a plausible scheme of the reaction involving nucleophilic attack of the hydride from Pt to the electrophilic carbon of the C=N bond of protonated oxime was proposed.

## Figures and Tables

**Table 1:** Data from the XRD analysis of the obtained oxide supports.

Sample	a, Å	a Table, Å	Phase	D, nm
CeO_2_-ZrO_2_	5.354	5.349	Ce_0.75_Zr_0.25_O_2_ Cerium Zirconium oxide	10
CeO_2_	5.410	5.411	CeO_2_ Cerianite	14
CeO_2_-ZrO_2_—n.u.	5.410	5.411	CeO_2_ Cerianite ZrO_2_ amorphous	10 n.d.

n.d.—not detected

**Table 2:** Textural properties of the prepared oxides and catalyst.

Sample	A_BET_ m^2^/g	V_total_^[a]^ cm^3^/g	V_meso_, cm^3^/g	V_micro, DFT_ cm^3^/g	D _av. pore_, nm
CeO_2_-ZrO_2_	70	0.162	0.158	0.006	8.9 (2–3, 3–5, 5–50)
1% Pt/CeO_2_-ZrO_2_	74	0.151	0.142	0.009	8.1 (1–2, 2–5, 5–50)
CeO_2_	58	0.121	0.109	0.009	8.0 (1–2, 2–15)
CeO_2_-ZrO_2_—n.u.	48	0.097	0.091	0.006	7.5 (2–5, 5–20)

^[a]^ Calculated from the adsorption value at *p*/*p*° = 0.99.

**Table 3:** H_2_ consumption by the reduced catalysts during the TPR-H_2_ analysis.

Sample	H_2_/Pt, mol/mol −100–25 °C	H_2_/Pt, mol/mol 24–300 °C
1% Pt/CeO_2_-ZrO_2_	8.3	4.6
1% Pt/CeO_2_	6.6	1.8
1% Pt/CeO_2_-ZrO_2_—n.u.	5.9	2.6
1% Pt/ZrO_2_	0.2	0
1% Pt/SiO_2_	0.2	0

**Table 4:** Activity of the Pt catalysts supported on different oxides in the hydrogenation of cyclohexanone oxime.

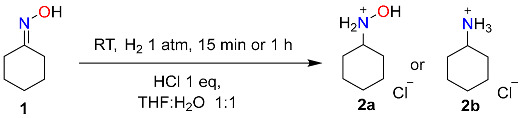						
№	Catalyst	Product	τ, h	X, %	Yield, % ^[a]^	TOF, h^−1 [b]^
1	1% Pt/ZrO_2_	2a	1	20	20	39
2	1% Pt/SiO_2_	2a	1	21	21	40
3	1% Pt/CeO_2_	2a	0.25	60	60	428
4	1% Pt/CeO_2_-ZrO_2_	2a	0.25	75	75	554
5	1% Pt/CeO_2_-ZrO_2_	2a	0.5	>99	>99	-
6	1% Pt/CeO_2_-ZrO_2_	2a	1	>99	>99	-
7	1% Pt/CeO_2_	2b	1	85	85	-
8	1% Pt/CeO_2_-ZrO_2_-n.u.	2a + 2b	1	>99	29 + 49	-
9 ^[c]^	1% Pd/AC-comm	-	2	0	0	-
10	1% Pt/C-comm	2a	4.5	68	48	-
11 ^[d]^	1% Pt/CeO_2_-ZrO_2_	2a	1	70	70	-

**Reaction conditions**: substrate (0.46 mmol), catalyst (50 mg, Pt 0.56% mol.), THF:H_2_O 1:1 vol. (2 mL), HCl (4.57 M, 80 µL), 15 min or 1 h, 25 °C, H_2_. ^[a]^ Calculated by ^1^H NMR using C_2_H_2_Cl_4_ as a standard; ^[b]^ TOF was calculated as the number of moles of the substrate reacted per number of moles of Pt in the catalyst per hour. ^[c]^ The commercial catalyst 1%Pd/AC was used (50 mg, Pd 0.47% mol), substrate (0.23 mmol), THF:H_2_O 1:1 vol. (2 mL), HCl (4.57 M, 40 µL), 25 °C, H_2_. ^[d]^ Substrate (3.68 mmol), catalyst (400 mg, Pt 0.56% mol.), THF:H_2_O 1:1 vol. (16 mL), HCl (4.57 M, 640 µL), 1 h, 25 °C, H_2_.

**Table 5:** Recycling of the 1% Pt/CeO_2_-ZrO_2_ catalyst after the first run.

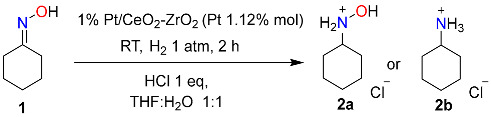			
Recycle	Product	X, %	Yield, % ^[a]^
1	2b	>99	>99
2	2b	>99	>99
3	2a	>99	>99

^[a]^ Isolated yield; X—conversion.

**Table 6:** Hydrogenation of ketoximes over the 1% Pt/CeO_2_-ZrO_2_ catalyst.

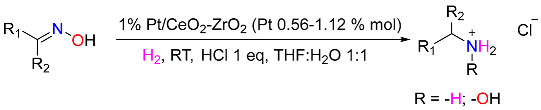						
№	Substrate	n Sub, mmol	Solvent, ml	τ, h	Product	Yield, % ^[a]^
1	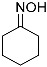 **1**	0.46	2	1	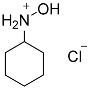 **2a**	91
2	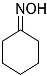 **1**	0.23	4	2	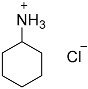 **2b**	99
3	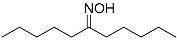 **3**	0.46	2	4.5	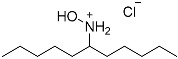 **4**	77
4	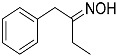 **5**	0.46	2	4.5	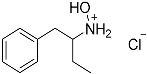 **6**	74
5	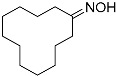 **7**	0.23	4	4.5	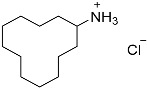 **8**	97
6	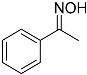 **9**	0.46	2	2	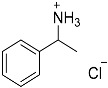 **10**	74
7	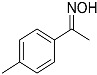 **11**	0.46	4	2	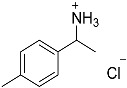 **12**	38 ^[b]^
8	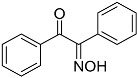 **13**	0.23	4	6	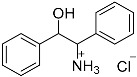 **14**	89

**Reaction conditions:** substrate (0.23 or 0.46 mmol), catalyst (50 mg, Pt 0.56–1.12% mol.), THF:H_2_O 1:1 vol., HCl (1 eq; 4.57 M,40 or 80 µL), 25 °C, H_2_. ^[a]^ Isolated yield. ^[b]^ Yield calculated by ^1^H NMR using C_2_H_2_Cl_4_ as a standard.

**Table 7:** Hydrogenation of aldoximes over the 1% Pt/CeO_2_-ZrO_2_ catalyst.

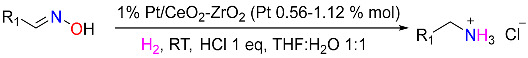						
№	Substrate	n Sub, mmol	Solvent, ml	τ, h	Product	Yield, % ^[a]^
1	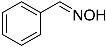 **15**	0.46	2	1	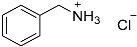 **16**	45 ^[b]^
2	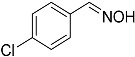 **17**	0.46	2	1	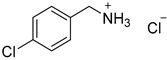 **18**	98
3	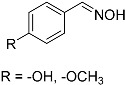 **19**	0.46	2	1	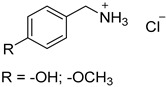 **20**	<5 ^[b]^
4	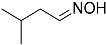 **21**	0.23	2	2	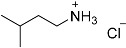 **22**	95
5	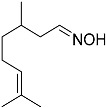 **23**	0.23	4	4	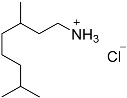 **24**	84
6	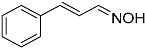 **25**	0.23	4	6	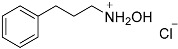 **26a** 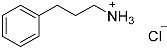 **26b**	70 ^[b]^ + 30 ^[b]^
7	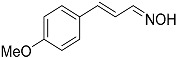 **27**	0.23	4	6	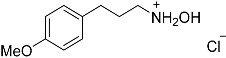 **28a** 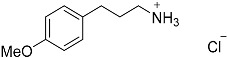 **28b**	55 ^[b]^ + 45 ^[b]^
8	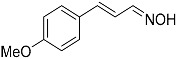 **27**	0.23	4	4	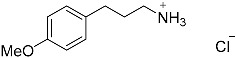 **28b**	48 ^[c]^

**Reaction conditions:** substrate (0.23 or 0.46 mmol), catalyst (50 mg, Pt 0.56–1.12% mol.), THF:H_2_O 1:1 vol., HCl (1 eq; 4.57 M,40 or 80 µL), 25 °C, H_2_. ^[a]^ Isolated yield. ^[b]^ Yield calculated by ^1^H NMR using C_2_H_2_Cl_4_ as a standard. ^[c]^ 40 °C.

## Data Availability

The data are contained within the article and Supplementary Materials.

## Supplementary Materials

The following supporting information can be downloaded at https://www.mdpi.com/article/10.3390/molecules30091926/s1: Figure S1. DRIFTS-CO study of the 1% Pt/ZrO_2_ sample; Figure S2. EDX-SEM map of element distribution of 1% Pt/CeO_2_-ZrO_2_ catalyst; Figure S3. SEM and TEM images of the used 1% Pt/CeO_2_-ZrO_2_ catalyst; Figure S4. EDX-SEM map of element distribution of the used 1% Pt/CeO_2_-ZrO_2_ catalyst; Table S1. Optimization of reaction conditions for cyclohexanone oxime hydrogenation; Figure S5. ^1^H and ^13^C NMR spectra of the reaction mixture obtained in cyclohexanone oxime hydrogenation for 4.5 h in THF:H_2_O in acidic media at 25 °C and H_2_ atmospheric pressure; Table S2. ICP-MS of fresh and used 1% Pt/CeO_2_-ZrO_2_ samples; ^1^H and ^13^C NMR spectra; HRMS for the products of hydrogenation of ketoximes; ^1^H and ^13^C NMR spectra for the products of hydrogenation of aldoximes; Table S3. Oximes prepared by mechanochemical synthesis. General procedure for the synthesis of oximes; additional references [74,75,76,77,78,79,80,81,82,83,84,85].
