# Supplementary material for: Ceria–Zirconia-Supported Pt as an Efficient Catalyst for the Sustainable Synthesis of Hydroxylamines and Primary Amines via the Hydrogenation of Oximes Under Ambient Conditions
Source: Molecules. 2025 Apr 26;30(9):1926. doi: 10.3390/molecules30091926 (PMC12074300; doi:10.3390/molecules30091926)
Supplement: Supplementary file 1 [file molecules-30-01926-s001.zip › molecules-3569253-supplementary.pdf]

Supporting Information

Ceria–Zirconia-Supported Pt as an Efficient Catalyst for the Sustainable Synthesis of Hydroxylamines and Primary Amines via the Hydrogenation of Oximes Under Ambient Conditions

Elena Redina *, Inna Ivanova, Olga Tkachenko, Gennady Kapustin, Igor Mishin and Leonid Kustov *

Laboratory of Development and Study of Polyfunctional Catalysts, N. D. Zelinsky Institute of Organic Chemistry of the Russian Academy of Sciences, 47 Leninsky Prospect, 119991 Moscow, Russia; inigiv022@gmail.com (I.I.); ot@ioc.ac.ru (O.T.); gik@ioc.ac.ru (G.K.); igo@ioc.ac.ru (I.M.)

* Correspondence: redinalena@yandex.ru or redinaea@ioc.ac.ru (E.R.); lmkustov@mail.ru (L.K.)

Table of contents

Figure S1. DRIFTS-CO study of the 1%Pt/ZrO_2 sample	S3
Figure S2. EDX-SEM map of element distribution of 1%Pt/CeO_2-ZrO_2 catalyst	S3
Figure S3. SEM and TEM images of the used 1%Pt/CeO_2-ZrO_2 catalyst	S4
Figure S4. EDX-SEM map of element distribution of the used 1%Pt/CeO_2-ZrO_2 catalyst	S4
Table S1. Optimization of reaction conditions for cyclohexanone oxime hydrogenation	S5
Figure S5. ^{1}H and ^{13}C NMR spectra of the reaction mixture obtained in cyclohexanone oxime hydrogenation for 4.5 h in THF:H_2O in acidic media at 25 ^{o}C and H_2 atmospheric pressure.	S6
Table S2. ICP-MS of fresh and used 1%Pt/CeO_2-ZrO_2 samples.	S7
^{1}H and ^{13}C NMR spectra, HRMS for the products of hydrogenation of ketoximes	S8-S15
^{1}H and ^{13}C NMR spectra for the products of hydrogenation of aldoximes	S16-S19
Table S3. Oximes prepared by mechanochemical synthesis. General procedure for the synthesis of oximes	S20
Description of ^{1}H and ^{13}C spectra, HRMS data for oximes obtained by mechanochemical synthesis	S21-S22
References	S23

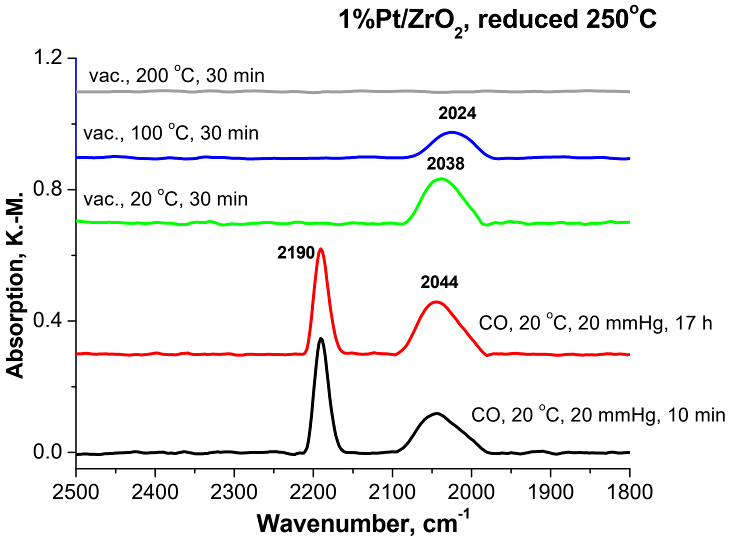

Figure S1. DRIFTS-CO study of the 1%Pt/ZrO_2 sample.

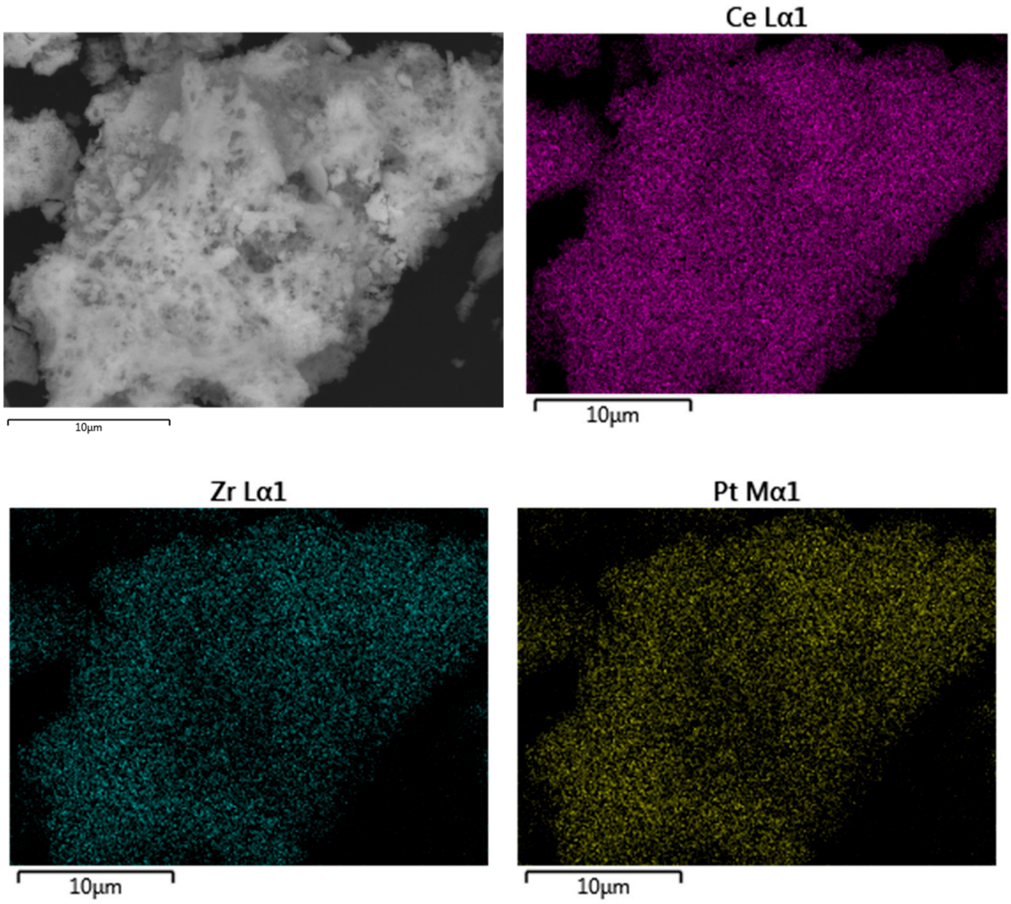

Figure S2. EDX-SEM map of element distribution of 1%Pt/CeO_2-ZrO_2 catalyst

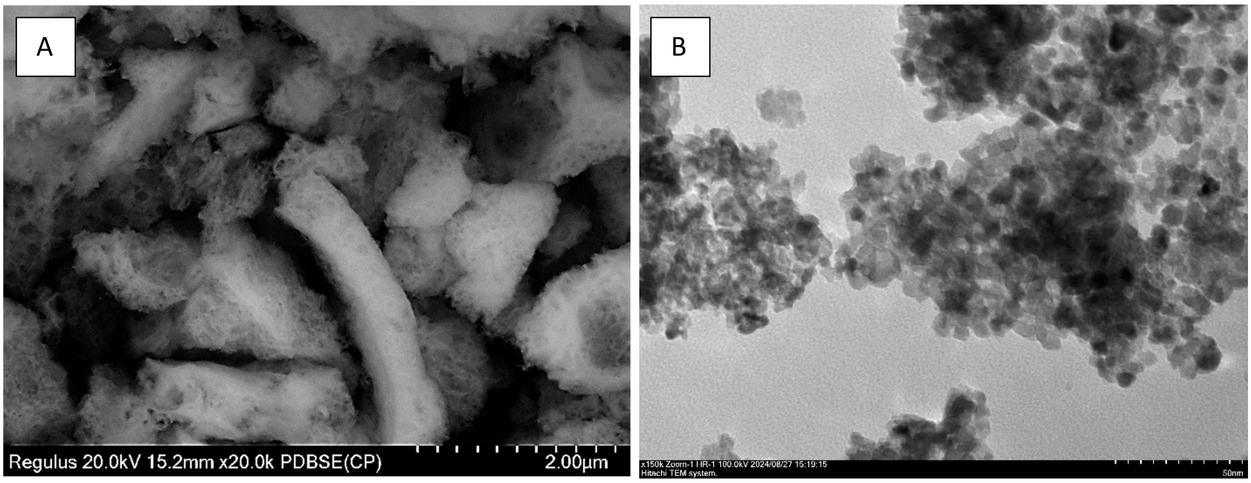

Figure S3. SEM (A) and TEM (B) images of the used 1%Pt/CeO_2-ZrO_2 catalyst

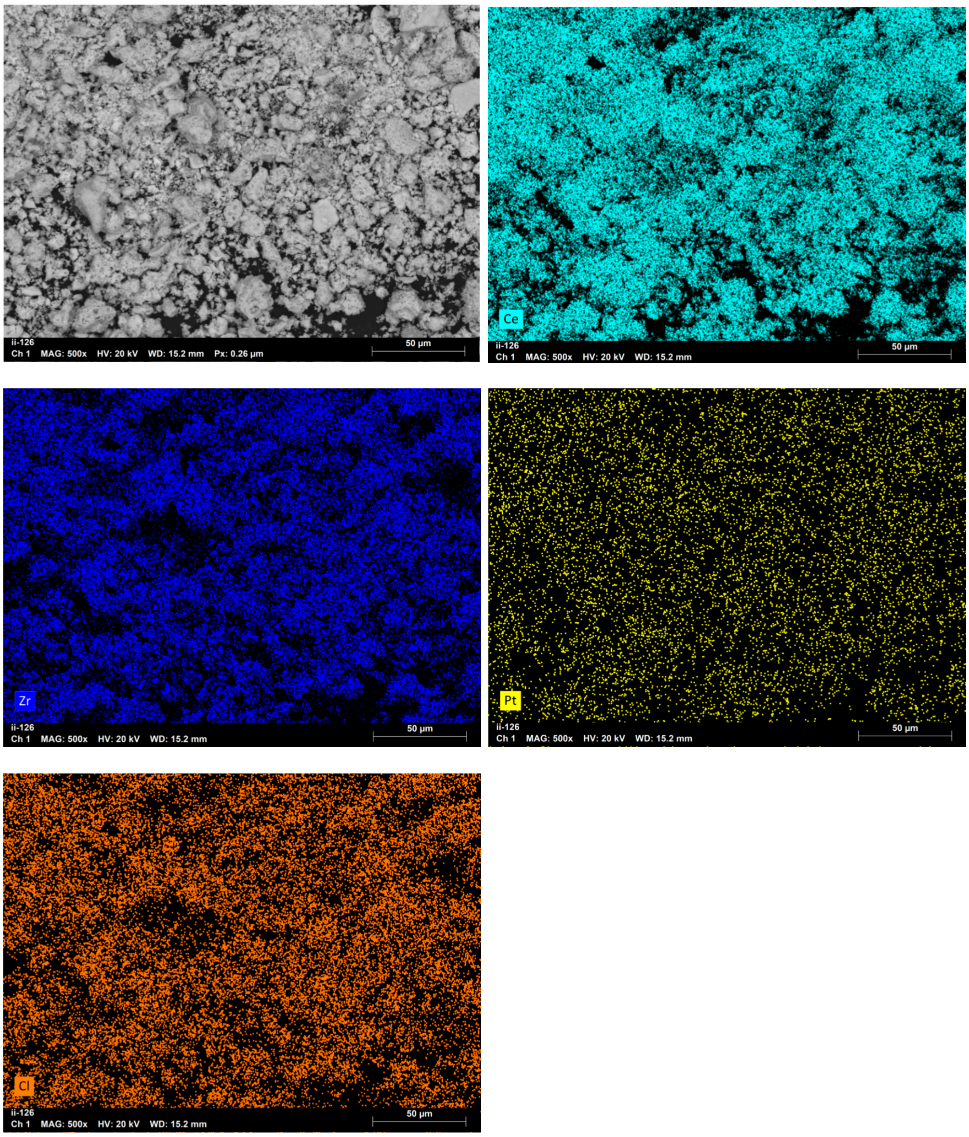

Figure S4. EDX-SEM map of element distribution of the used 1%Pt/CeO_2-ZrO_2 catalyst

Table S1. Optimization of reaction conditions for cyclohexanone oxime hydrogenation.

№	Substrate	N sub, mmol	Solvent	t, °C	H_2, atm	V HCl, µl	τ, h	X, %	Product [a]
1		0.46	THF	60	20	no	4.5	no	no
2		0.46	THF	60	20	80	4.5	35	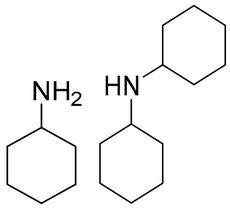 **2b** **2c** 50:50
3		0.46	THF+H_2O 1:1	60	20	80	4.5	>99	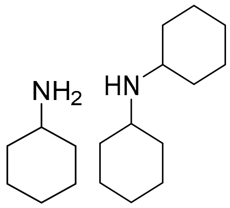 **2b** **2c** 50:50
4	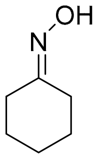 **1**	0.46	THF+H_2O 1:1	25	1	80	4.5	>99	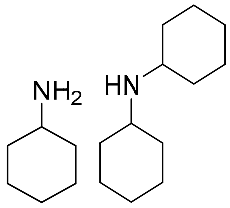 **2b** **2c** 55:45
5		0.46	THF+H_2O 1:1	25	1	no	4.5	no	no
6		0.23	THF+H_2O 1:1	25	1	80	4.5	>99	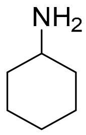 **2b**
7		0.23	THF+H_2O 1:1	25	1	80	2	>99	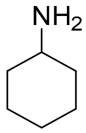 **2b**
8		0.46	THF+H_2O 1:1	25	1	80	1	>99	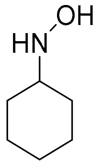 **2a**

[a] Determined by ^{1}H and ^{13}C NMR with $C_2H_2Cl_4$ used as an external standard.

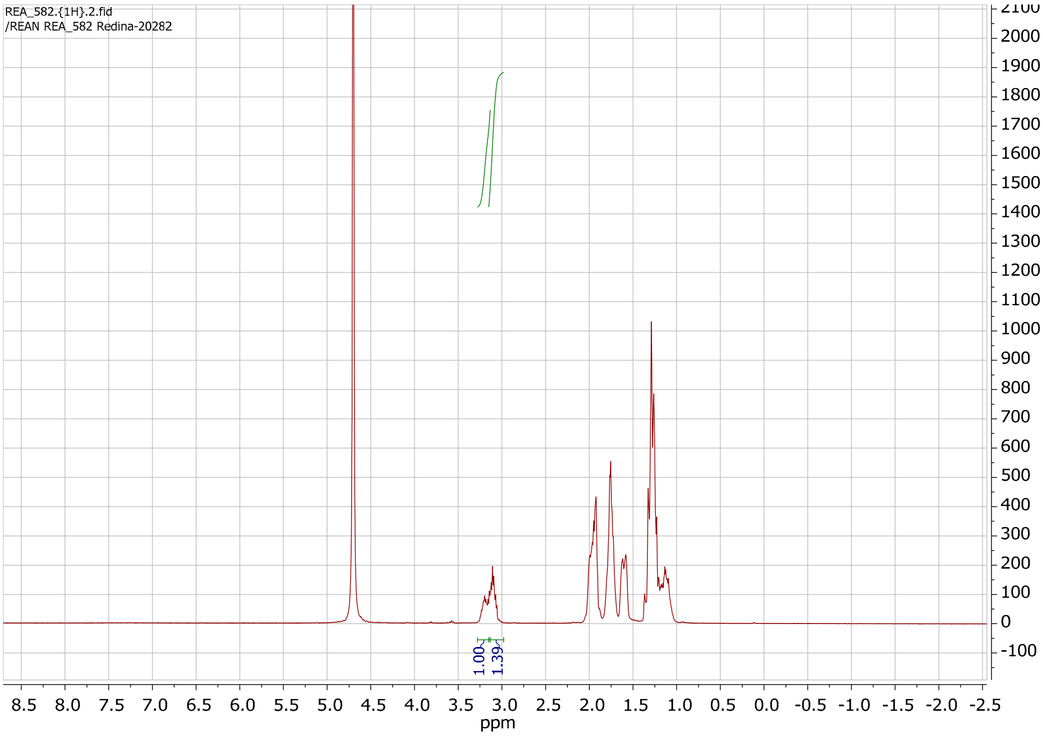

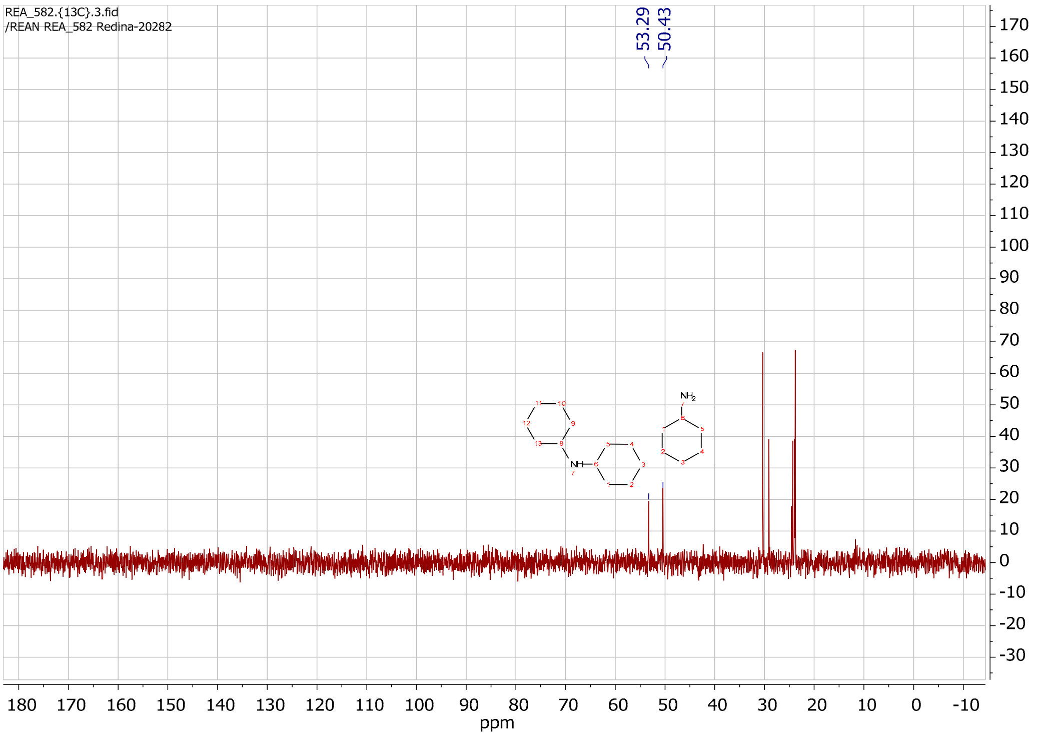

Figure S5. ^{1}H and ^{13}C NMR spectra in D_2O of the reaction mixture obtained in cyclohexanone oxime (0.46 mmol) hydrogenation for 4.5 h in THF:H_2O (1 ml: 1 ml) in acidic media (HCl 4.57 aq, 80 µl) at 25 ^{o}C and H_2 atmospheric pressure. The assignment of signals was made according to "Integrated Spectral Data Base System of Organic Compounds" (data were obtained from the National Institute of Advanced Industrial Science and Technology (Japan)).

Table S2. ICP-MS of fresh and used 1%Pt/CeO_2-ZrO_2 samples.

Sample	Pt, % wt.
1%Pt/CeO_2-ZrO_2	0.98
1%Pt/CeO_2-ZrO_2 used for 4 cycles	0.84

The actual Pt content was obtained via inductively coupled plasma mass spectrometry (ICP-MS) using an iCAP Q instrument (Thermo Scientific). Before analysis, Pt/CeO_2-ZrO_2 catalysts were previously dissolved in Aqua Regia (1 mg of the sample per 2.4 ml of acid) using a microwave digestion system (200 ^{o}C, 1200 W, 1 hour).

Hydrogenation of ketoximes

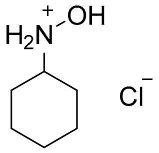

N-cyclohexylhydroxylammonium chloride (**2a**). White solid, yield 91% (63 mg).

^{1}H NMR (300 MHz, D_2O), δ: 3.31-3.21 (m, 1H), 2.01-1.14 (m, 12H).

^{13}C NMR (75 MHz, D_2O), δ: 60.35, 26.38, 24.52, 23.47.

HRMS: m/z calculated for $C_6H_{15}NO^+$ 116.1070, found 116.1068.

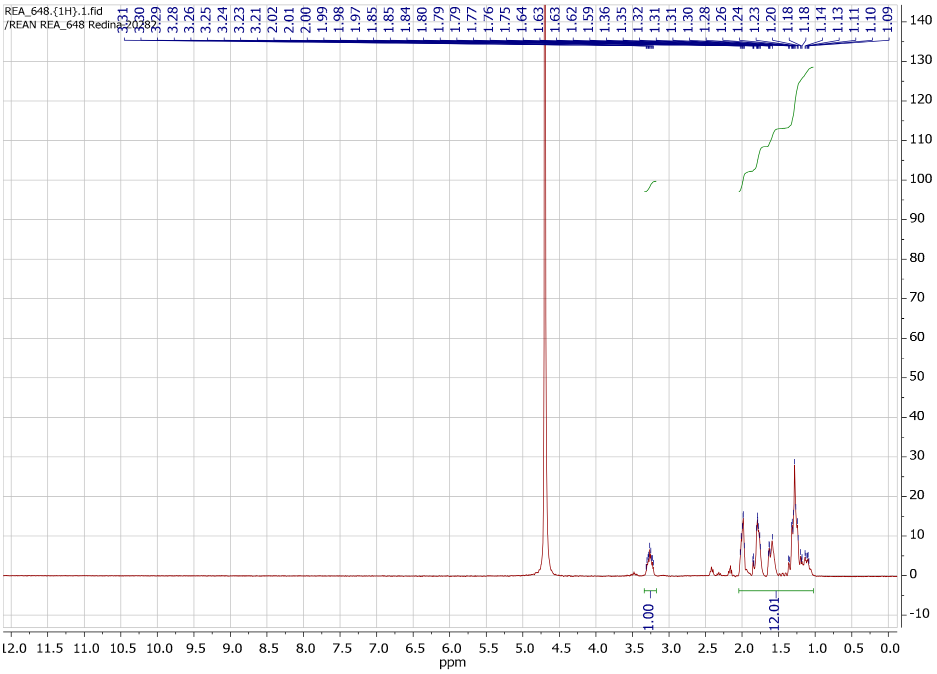

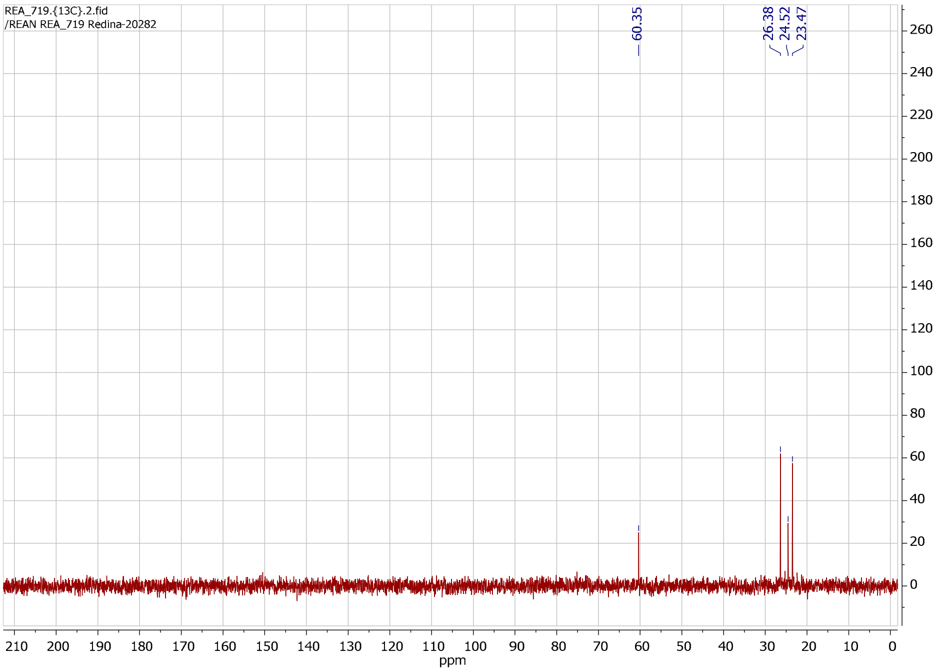

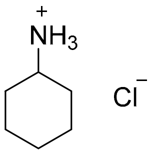

Cyclohexylamine hydrochloride **(2b)** [1]. White solid, yield 99% (37 mg).

^{1}H NMR (300 MHz, D_2O), δ: 3.23-3.13 (m, 1H, CH), 2.02-1.20 (m, 11H).

^{13}C NMR (75 MHz, D_2O), δ: 50.41, 30.34, 24.30, 23.81.

HRMS: m/z calculated for $C_6H_{14}N^+$ 100.1121, found 100.1123.

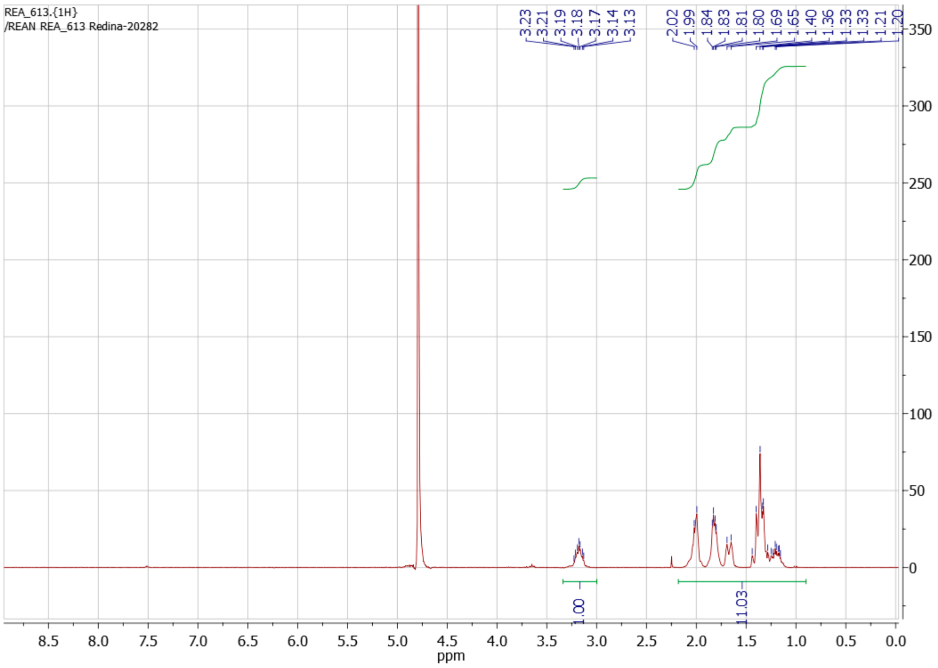

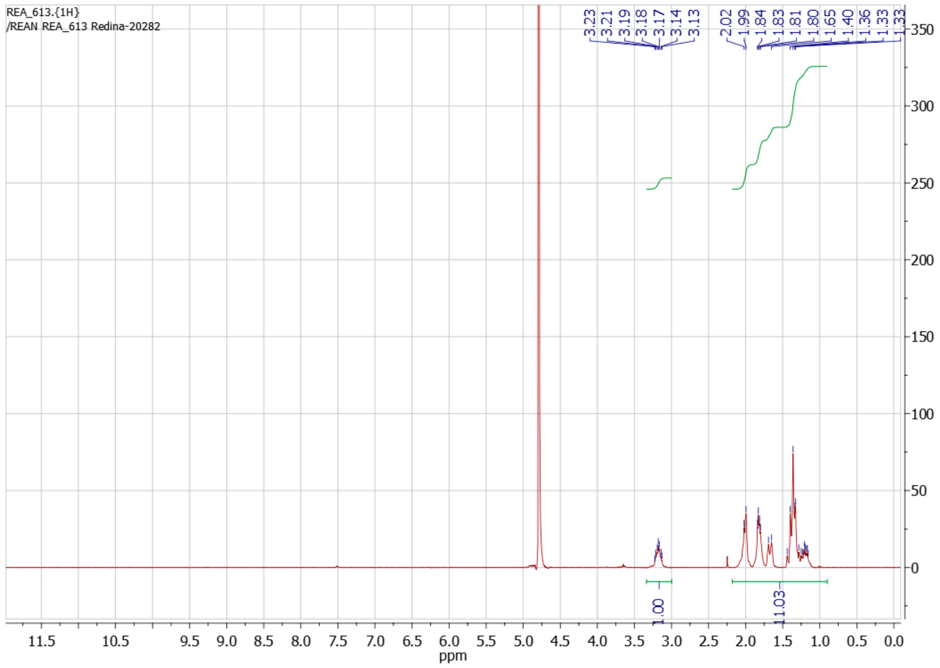
REA_613.{1H}
/REAN REA_613 Redina-20282
3.23
3.21
3.19
3.18
3.17
3.14
3.13
2.02
1.99
1.84
1.83
1.81
1.80
1.65
1.40
1.36
1.33
1.33
1.00
11.03
ppm

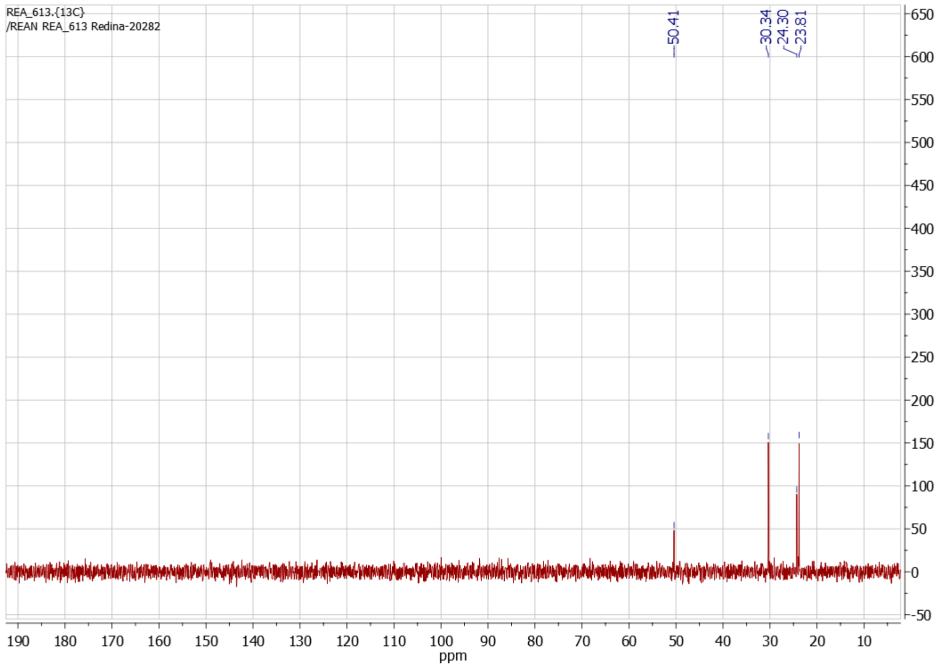
REA_613.{13C}
/REAN REA_613 Redina-20282
50.41
30.34
24.30
23.81
ppm

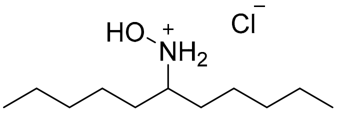

N-(Undec-6-yl)hydroxylammonium chloride **(4)**. Pale yellow solid, 77% (79 mg).

^{1}H NMR (300 MHz, D_2O), δ: 3.37-3.28 (m, 1H), 1.64-1.61 (m, 4H), 1.33-1.25 (m, 12H), 0.81 (t, *J* = 6.7 Hz, 6H).

^{13}C NMR (75 MHz, D_2O), δ: 61.96, 30.77, 27.78, 23.90, 21.66, 13.20.

HRMS: m/z calculated for $C_{11}H_{26}NO^+$ 188.2009, found 188.2009.

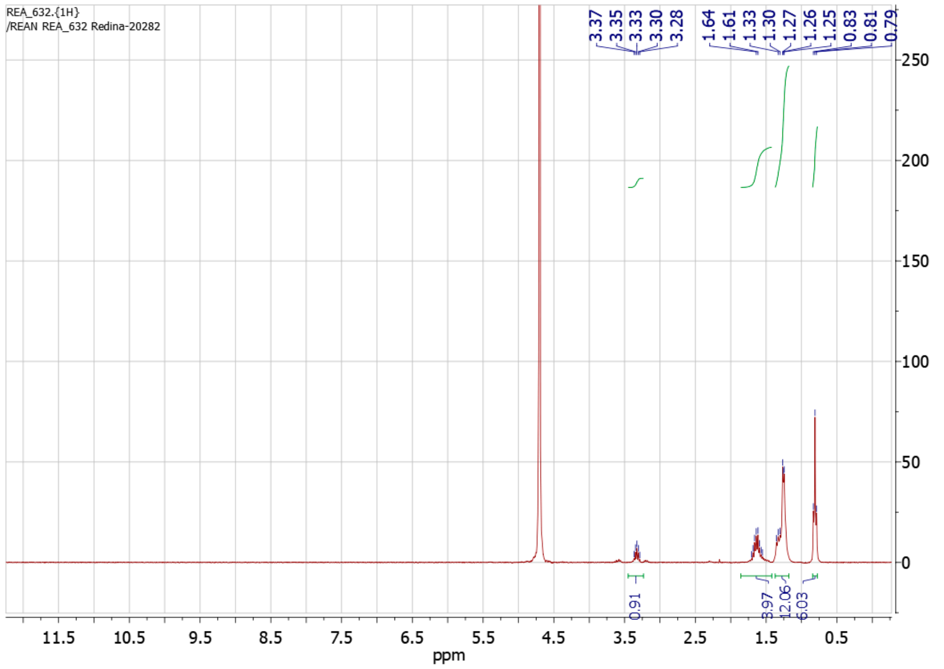

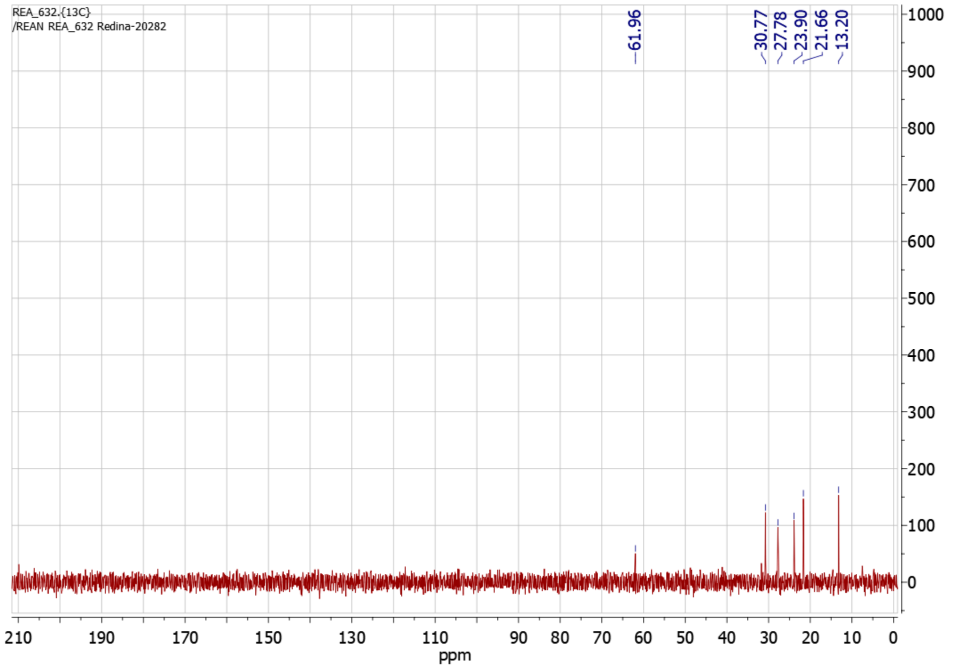

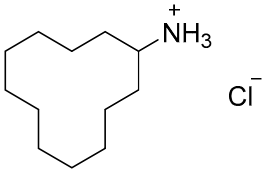

Cyclododecylammonium chloride **(6a)** [1]. White solid, 89% (45 mg).

^{1}H NMR (300 MHz, DMSO-d_6), δ: 8.19 (br s, 3H), 3.09 (s, 1H), 1.63-1.22 (m, 22H).

^{13}C NMR (75 MHz, DMSO-d_6), δ: 47.87, 27.53, 23.61, 23.31, 22.91, 22.83, 20.35.

HRMS: m/z calculated for $C_{12}H_{26}N^{+}$ 184.2060, found 184.2066.

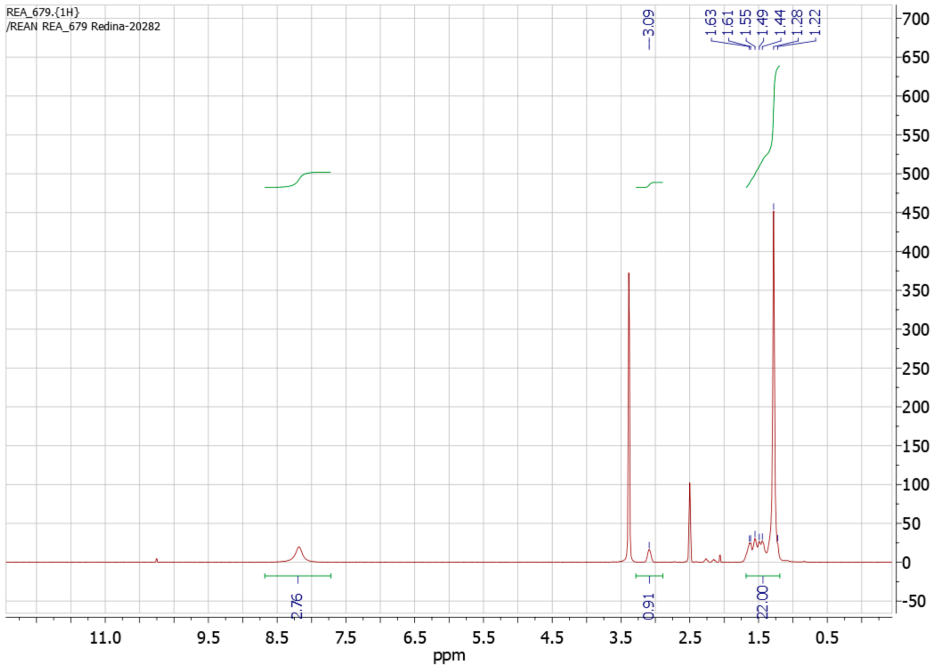

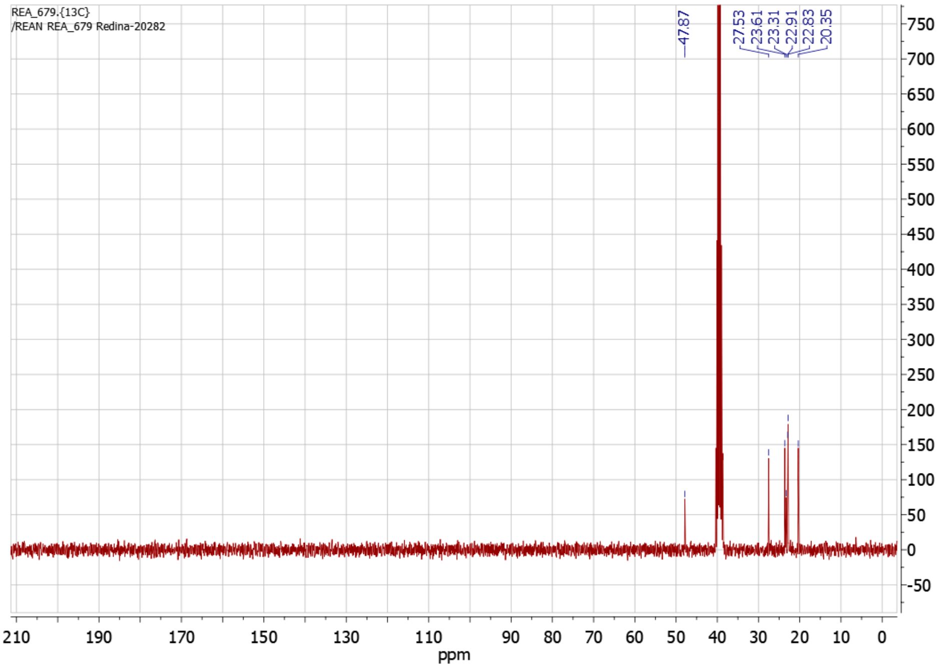

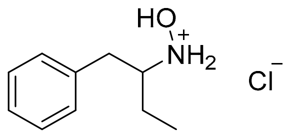

N-(1-Phenylbut-2-yl)hydroxylammonium chloride **(8)** [2]. White solid, yield 74% (73 mg).

^{1}H NMR (300 MHz, D_2O), δ: 7.51 – 7.25 (m, 5H), 3.60 (dt, *J* = 13.3, 6.7 Hz, 1H), 3.01 (dd, *J* = 7.0, 3.2 Hz, 2H), 1.78 – 1.60 (m, 2H), 0.95 (t, *J* = 7.5 Hz, 3H).

^{13}C NMR (75 MHz, D_2O), δ: 138.91, 129.51, 129.02, 127.42, 64.16, 33.84, 21.07, 8.83.

HRMS: m/z calculated for $C_{10}H_{16}NO^{+}$ 166.1226, found 166.1231.

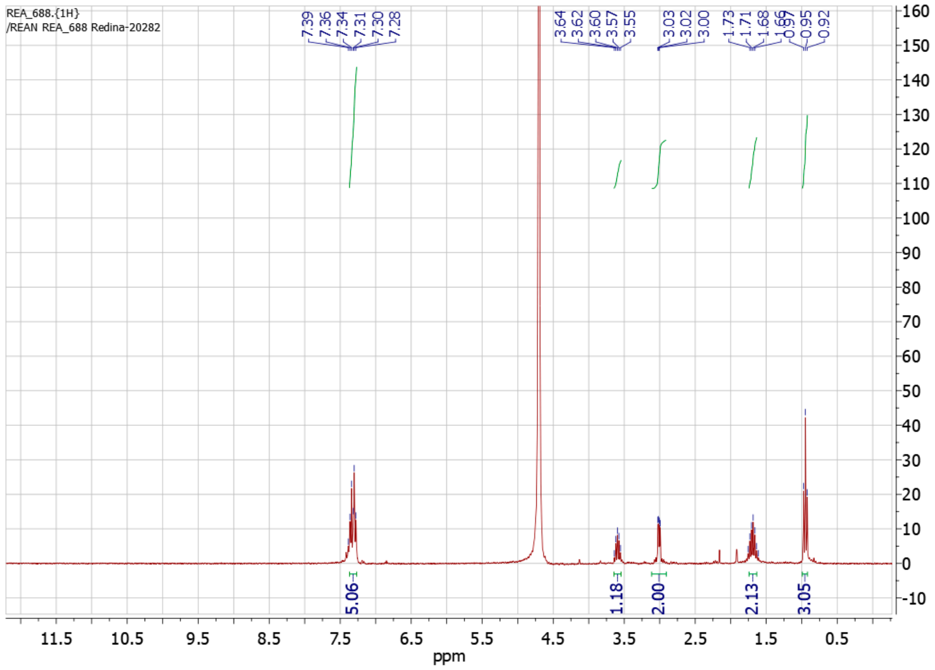

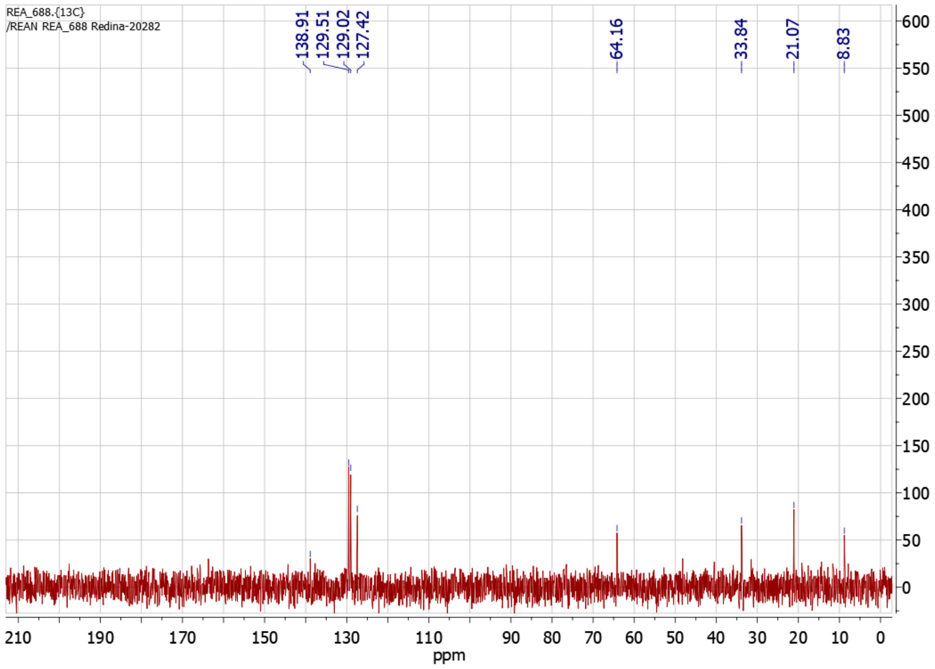

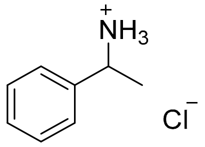

α-Methylbenzylamine hydrochloride **(10)** [1]. White solid, yield 74% (54 mg).

^{1}H NMR (300 MHz, D_2O), δ: 7.45-7.39 (m, 5H), 4.49 (q, J = 6.9 Hz, 1H), 1.58 (d, J = 6.9 Hz, 3H).

^{13}C NMR (75 MHz, D_2O), δ: 137.74, 129.27, 129.17, 126.54, 51.04, 19.33.

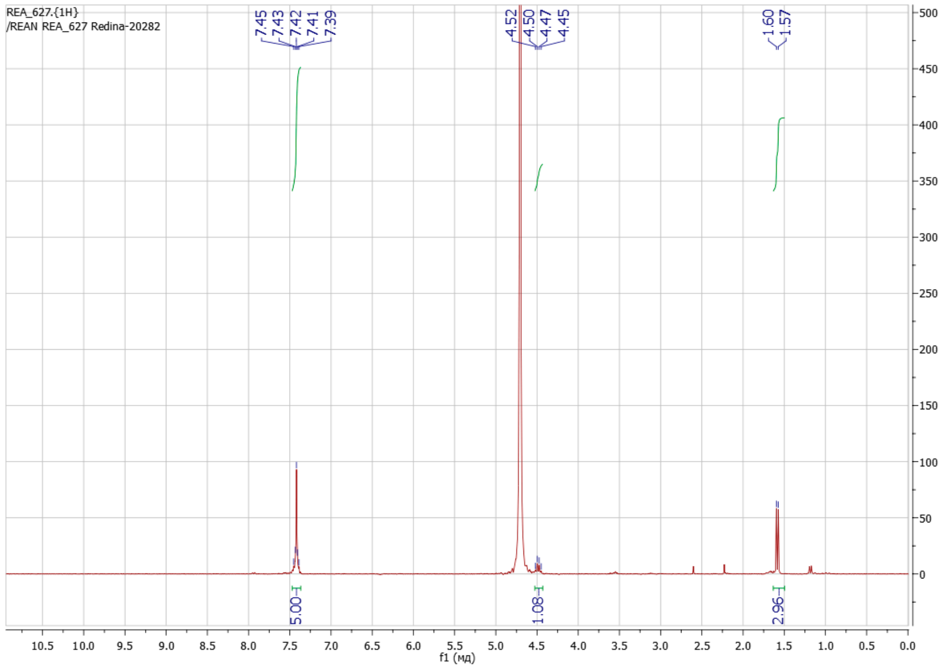

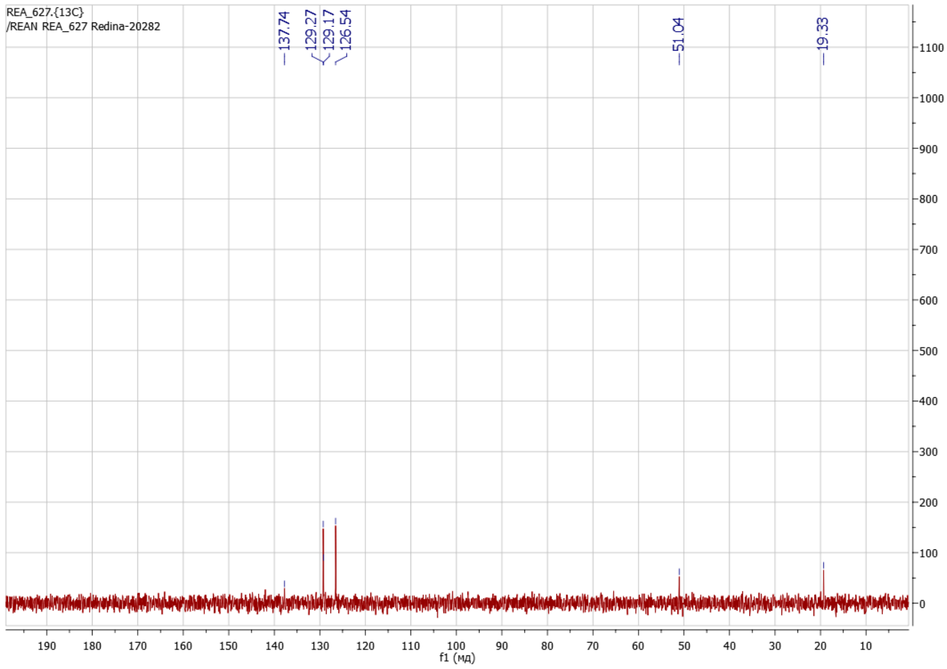

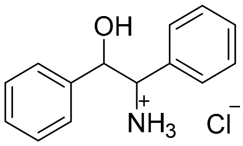

2-Hydroxy-1,2-diphenylethylammonium chloride **(14)** [3]. White solid, 89% (61 mg).

^{1}H NMR (300 MHz, DMSO-*d*$_6$), δ: 8.82 (s, 3H), 7.26 – 7.06 (m, 10H), 6.20 (d, *J* = 4.0 Hz, 1H), 5.33 (s, 1H), 4.38 (d, *J* = 3.3 Hz, 1H).

^{13}C NMR (75 MHz, DMSO-*d*$_6$), δ: 140.89, 133.63, 129.06, 128.13, 127.75, 127.54, 127.26, 126.26, 72.09, 59.85.

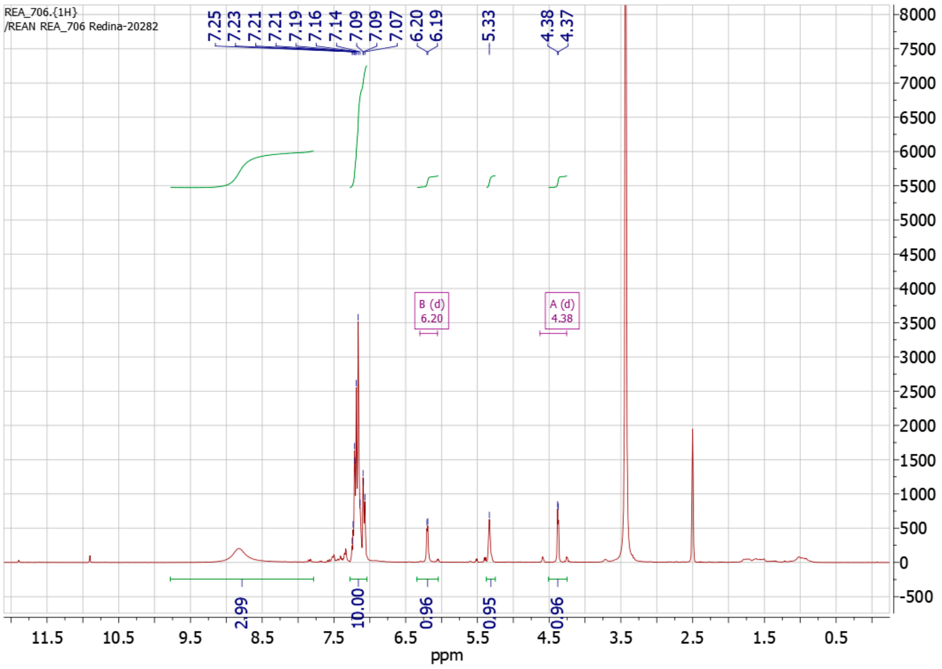

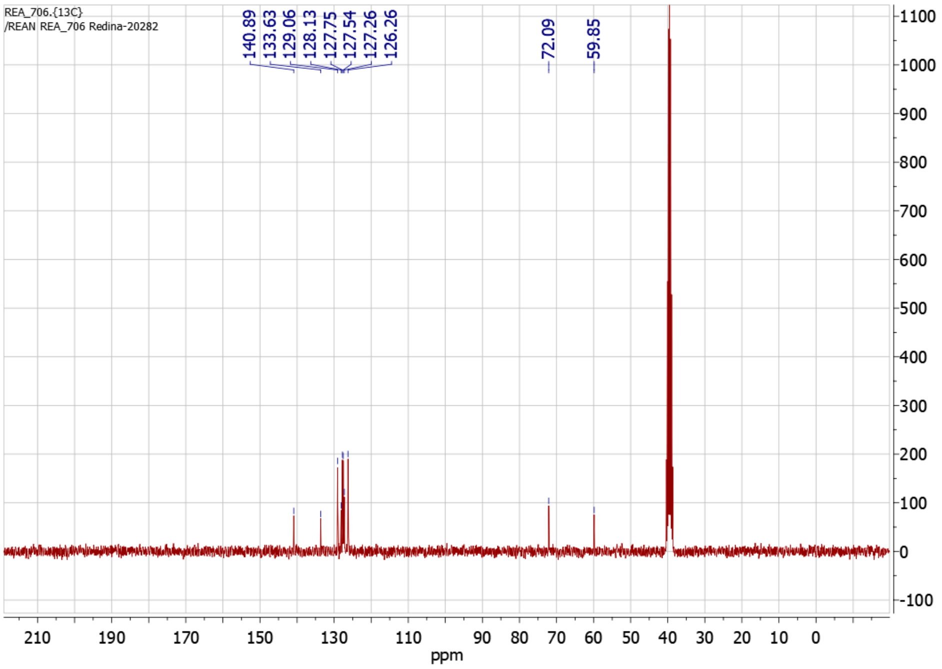

Hydrogenation of aldoximes

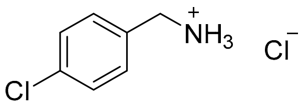

p-Chlorobenzylammmonium chloride **18** [4]**.** White solid, 98% (67 mg).

^{1}H NMR (300 MHz, D_2O), δ: 7.48 (d, *J* = 8.6 Hz, 2H), 7.41 (d, *J* = 8.6 Hz, 2H), 4.16 (s, 2H).

^{13}C NMR (75 MHz, D_2O), δ: 131.20, 130.44, 130.38, 129.17, 42.43.

HRMS (ESI) m/z calculated for [C7H9ClN]+ [M]+: 142.0418, found 142.0426.

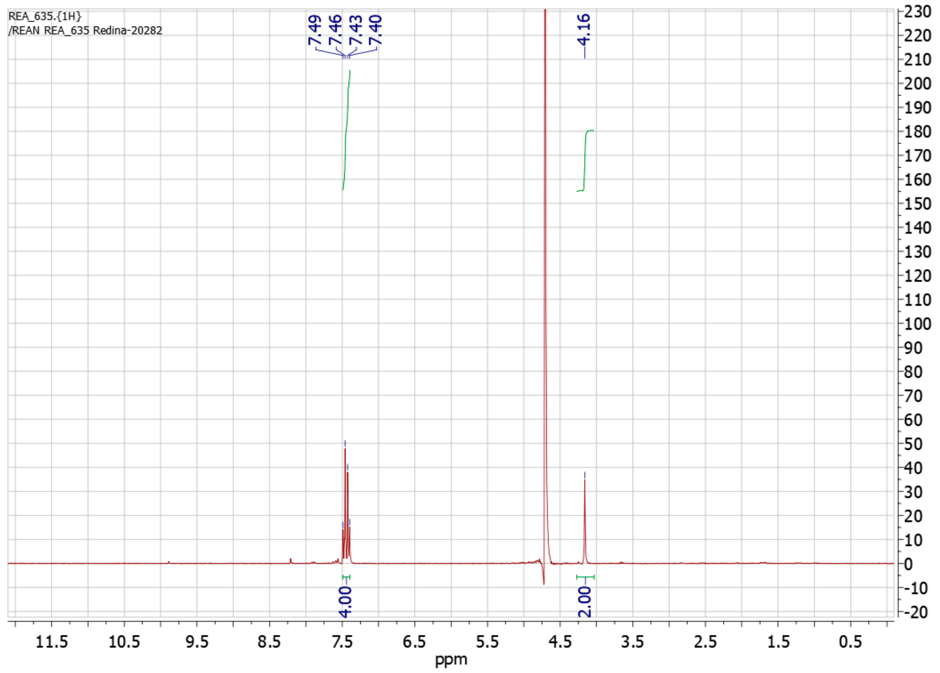

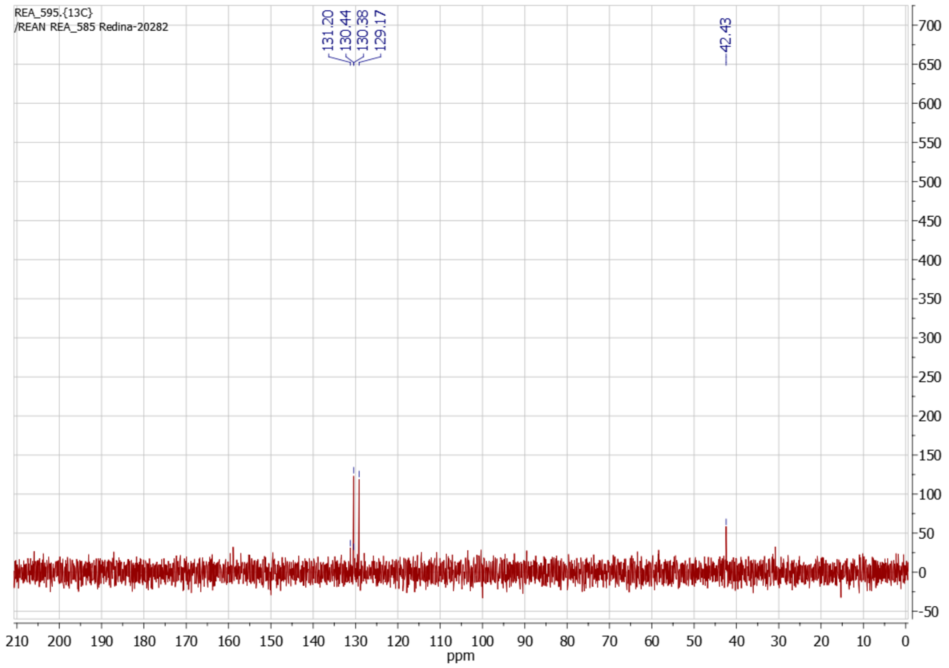

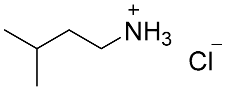

3-Methylbutylammonium chloride **22** [5]. White solid, 95% (33 mg).

^{1}H NMR (300 MHz, D_2O), δ: 2.90-2.82 (m, 2H), 1.51-1.44 (m, 2H), 1.43 – 1.32 (m, 2H), 0.74 (d, *J* = 6.4 Hz, 6H)

^{13}C NMR (75 MHz, D_2O), δ: 46.03, 34.21, 25.25, 21,37.

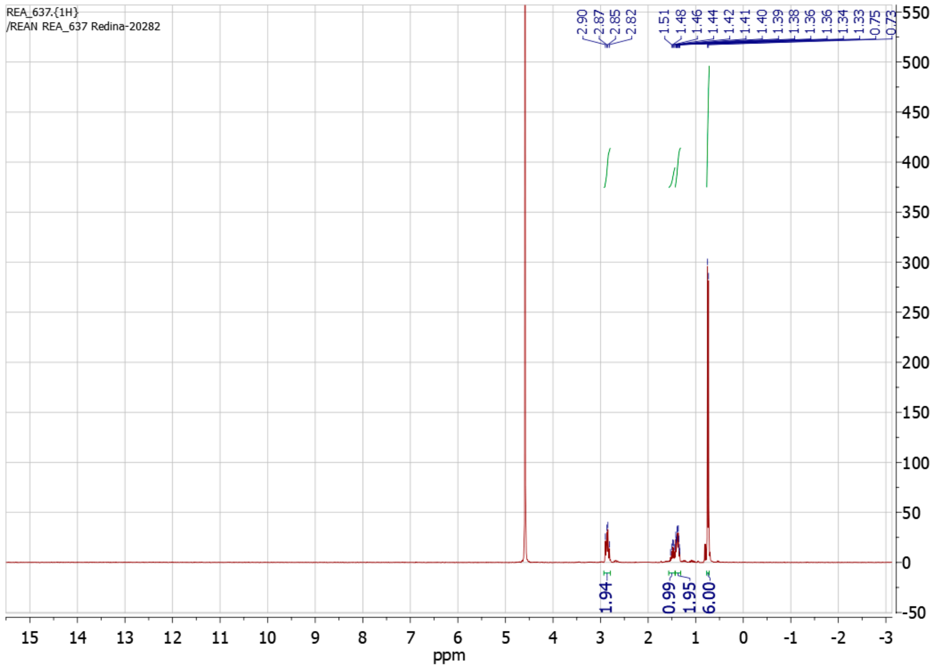

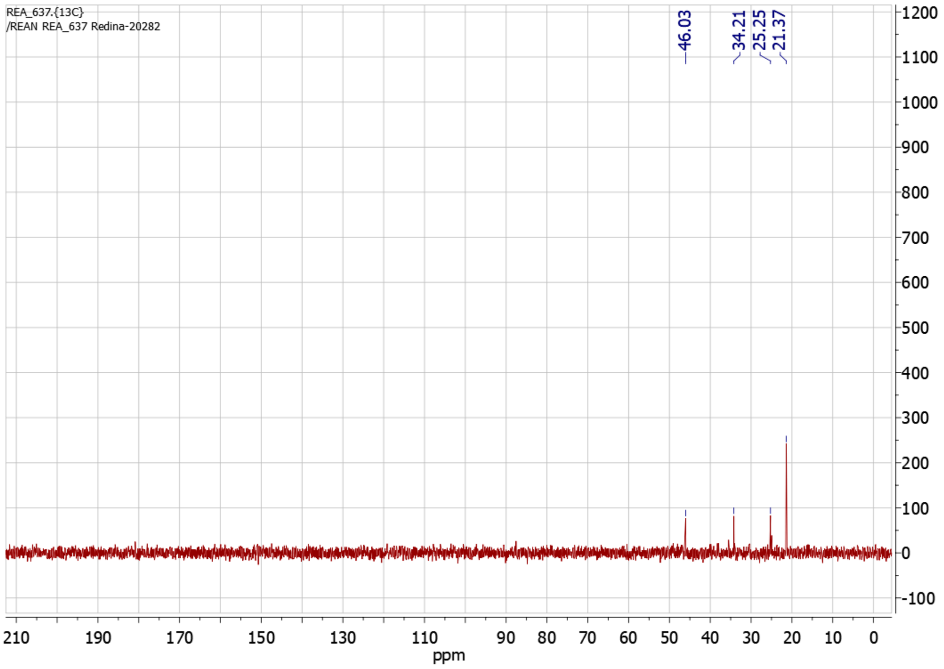

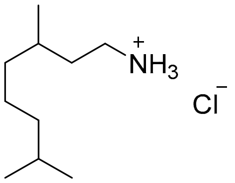

3,7-Dimethyloctylammonium chloride **24** [6]. Yellow liquid, 84% (37 mg)

^{1}H NMR (300 MHz, DMSO-d6), δ: 2.61-2.76 (m, 2H), 1.61-1.02 (m, 12H), 0.81 (d, *J* = 6.4 Hz, 9H)

^{13}C NMR (75 MHz, DMSO-d6), δ: 38.79, 37.08, 36.52, 33.88, 29.96, 27.51, 24.07, 22.73, 22.63, 19.37.

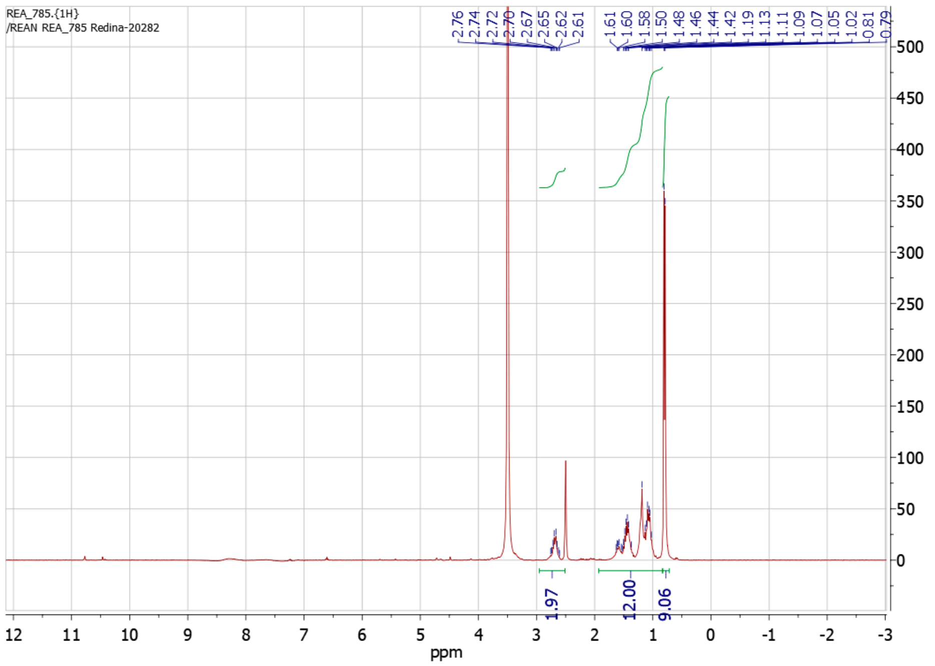

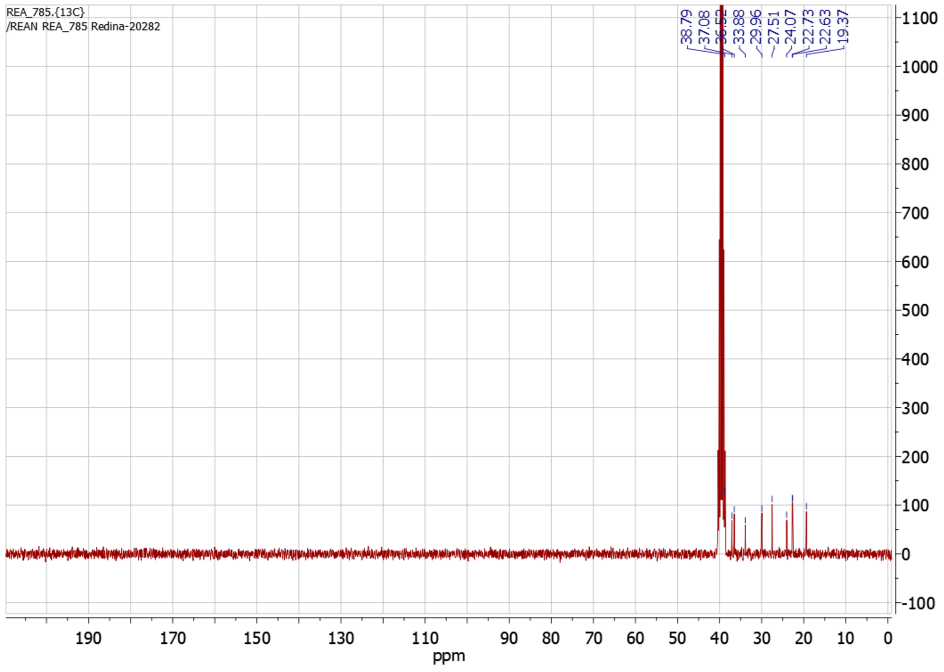

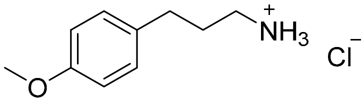

3-(4-Methoxyphenyl)propylammonium chloride **28b** [7]. White solid, 48% (53 mg).

^{1}H NMR (300 MHz, DMSO-d6), δ: 7.09 (d, J = 8.5 Hz, 1H), 6.79 (d, J = 8.6 Hz, 1H), 3.63 (s, 2H), 2.72 – 2.61 (m, 2H), 2.60 – 2.48 (m, 2H), 1.90-1.77 (m, 2H).

^{13}C NMR (75 MHz, DMSO-d6), δ: 157.63, 132.94, 129.38, 113.93, 55.16, 38.29, 31.16, 28.93.

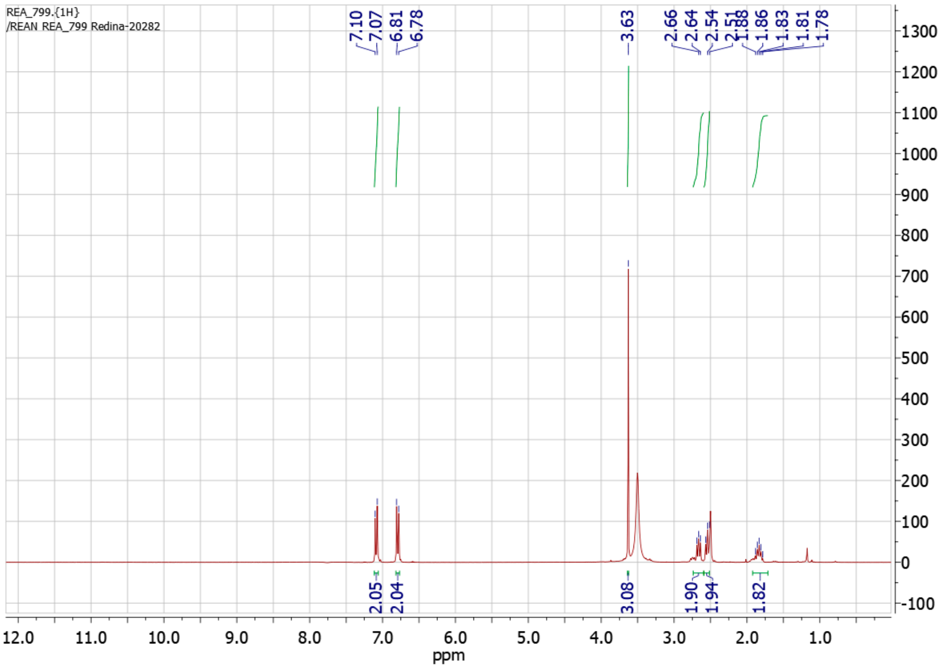

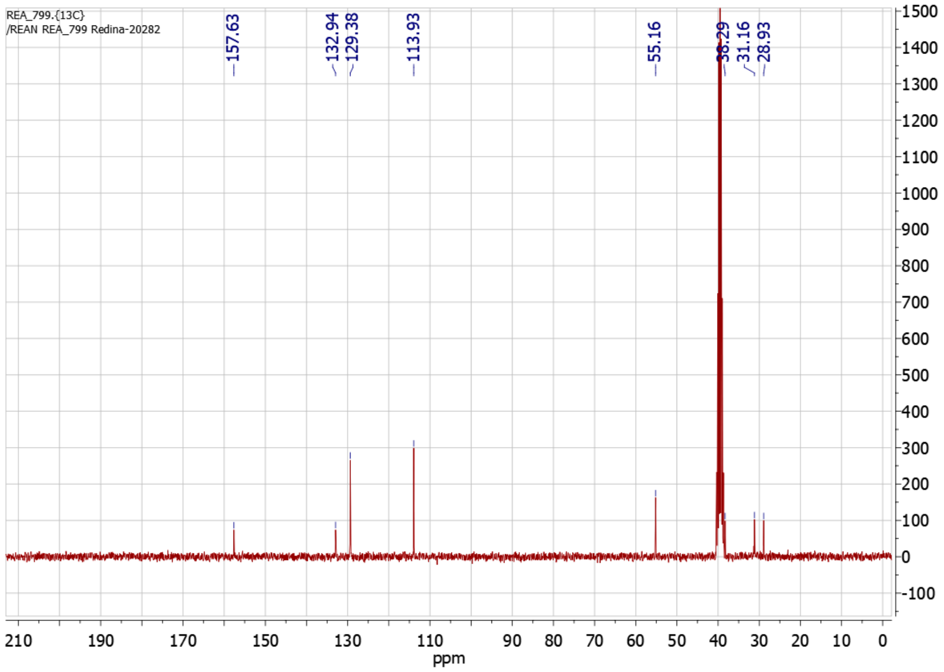

Oxime synthesis

Table S3. Oximes prepared by mechanochemical synthesis.

№	Product	t, min	Yield, %
1	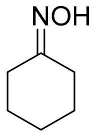	10	75
11	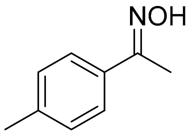	30	45
17	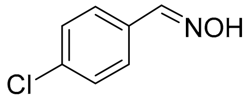	15	71
19a	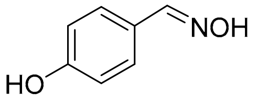	15	61
19b	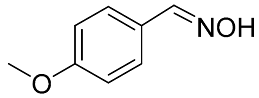	12	55
23	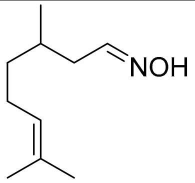	12	60
25	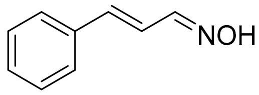	15	75
27	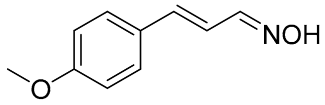	12	73

General procedure:

A mixture of a carbonyl compound, hydroxylamine hydrochloride, and sodium hydroxide in a molar ratio 1:1.2:1.2, respectively, was ground in a mortar with a pestle for 10 min for 5 times during 1 h until the reaction completed. For ketones, 0.4 g of silica gel per 1 mmol of a substrate was also added to the reaction mixture. The reaction was monitored by TLC. Upon completion of the reaction, the reaction mixture was washed with deionized H_2O to get rid of inorganic salts and then dried under a vacuum. In case of liquid products or ketones, the reaction mixture was extracted with 2*10 ml of ethyl acetate, then washed with deionized H_2O, dried over Na_2SO_4 and evaporated under a vacuum. The substances were characterized by ^1H, ^{13}C NMR and HRMS, if necessary.

Cyclohexanone oxime [8]

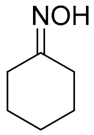

White solid, yield 75% (754 mg)

^{1}H NMR (300 MHz, DMSO-d_6), δ: 10.09 (s, 1H, NOH), 2.42 – 2.31 (m, 2H, CH_2), 2.16 – 2.06 (m, 2H, CH_2), 1.63 – 1.43 (m, 6H, CH_2).
^{13}C NMR (75 MHz, DMSO-d_6), δ: 157.62, 32.08, 27.16, 25.91, 25.75, 24.32.

1-(p-Tolyl)ethan-1-one oxime [9]

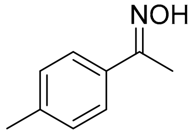

White solid, yield 45% (190 mg)

^{1}H NMR (300 MHz, DMSO-d_6), δ: 11.07 (s, 1H), 7.53 (d, J = 8.2 Hz, 2H), 7.18 (d, J = 8.1 Hz, 2H), 2.30 (s, 3H), 2.12 (s, 3H).

^{13}C NMR (75 MHz, DMSO-d_6), δ: 153.22, 138.46, 134.69, 129.38, 125.91, 21.23, 11.95.

4-Chlorobenzaldehyde oxime [9]

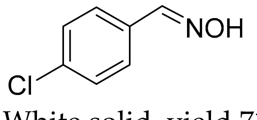

White solid, yield 71% (110 mg)

^{1}H NMR (300 MHz, DMSO-d_6), δ: 11.36 (s, 1H), 8.15 (s, 1H), 7.61 (d, J = 8.5 Hz, 2H), 7.46 (d, J = 8.5 Hz, 2H).

^{13}C NMR (75 MHz, DMSO-d_6), δ: 147.60, 134.18, 132.48, 129.26, 128.51.

4-Hydroxybenzaldehyde oxime [10]

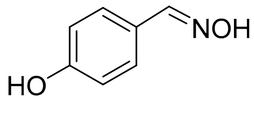

White solid, yield 61% (84 mg)

^{1}H NMR (300 MHz, DMSO-d_6), δ: 10.83 (s, 1H), 8.01 (s, 1H), 7.41 (d, J = 8.5 Hz, 2H), 6.78 (d, J = 8.4 Hz, 2H).

^{13}C NMR (75 MHz, DMSO-d_6), δ: 158.99, 148.35, 128.41, 124.48, 116.00.

E,Z-4-Methoxybenzaldehyde oxime [10]

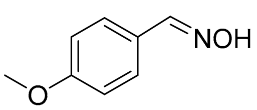

Yellow oil, yield 55% (247 mg)

^{1}H NMR (300 MHz, DMSO-d_6), δ: 11.36 (s, 0.12H), 10.95 (s, 0.88H), 0.88 (s, 0.88H), 7.94 (d, J = 8.9 Hz, 0.24H), 7.52 (d, J = 8.8 Hz, 1.76H), 7.31 (s, 0.12H), 7.01-6.91 (m, 2H), 3.79 (s, 0.36H), 3.77 (s, 2.64H).

^{13}C NMR (75 MHz, DMSO-d_6), δ: 160.12, 147.66, 132.30, 127.85, 125.63, 114.20, 113.68, 55.20.

E,Z-3,7-Dimethyloct-6-enal oxime [11]

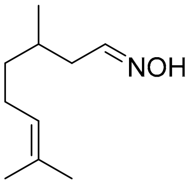

Colorless oil, yield 60% (510 mg)

^{1}H NMR (300 MHz, DMSO-d_6), δ: 10.70 (s, 0.48H), 10.37 (2, 0.52H), 7.28 (t, J = 6.3 Hz, 0.52H), 6.65 (t, J = 5.4 Hz, 0.48H), 5.19-5.00 (m, 1H), 2.37-0.99 (m, 7H), 1.64 (s, 3H), 1.56 (s, 3H), 0.98-0.76 (m, 3H).

^{13}C NMR (75 MHz, DMSO-d_6), δ: 149.69, 149.03, 131.09, 124.91, 124.87, 40.83, 40.56, 40.28, 40.00, 39.72, 39.45, 39.17, 36.91, 36.67, 36.42, 32.02, 30.87, 30.47, 25.95, 25.47, 25.39, 20.04, 19.79, 17.95.

HRMS : m/z calculated for $[M+H]^+$ $C_{10}H_{19}NO$ 170.1539; found 170.1540.

Cinnamaldehyde oxime [12]

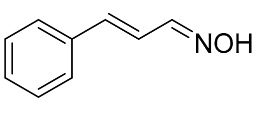

Pale yellow solid, yield 75% (517 mg)

^{1}H NMR (300 MHz, DMSO-d_6), δ: 11.25 (s, 1H); 7.57-7.31 (m, 6H, ArH, CH); 6.96 (m, 1H, CH).

^{13}C NMR (75 MHz, DMSO-d_6), δ: 147.21; 137.87; 135.91; 129.02; 128.88; 127.19; 116.14.

HRMS: m/z calculated for C_9H_9NO $[M+H]^+$ 148.0757; found 148.0758.

E,Z-4-Methoxycinnamaldehyde oxime [12]

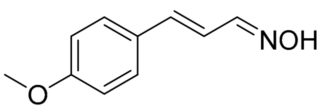

Yellow solid, yield 73% (258 mg)

^{1}H NMR (300 MHz, DMSO-d_6), δ: 11.09 (s, 0.64H), 10.95 (s, 0.36H), 7.86 (d, J = 8.5 Hz, 0.36H), 7.57-7.42 (m, 2H), 7.29-7.12 (m, 1.28H), 7.00-6.89 (m, 2.36H), 6.89-6.68 (m, 1H), 3.775 (s, 1.92H), 3.765 (s, 1.08H) ^{13}C NMR (75 MHz, DMSO-d_6), δ: 160.02; 159.54; 150.45; 147.50; 137.65; 136.39; 128.74; 128.53; 128.19; 120.71; 114.37; 114.25; 114.10; 55.23; 55.18.

HRMS: m/z calculated for $C_{10}H_{11}NO_2$ $[M+H]^+$ 178.0863; found 178.0868.

References

[1] Q. An, Z. Wang, Y. Chen, X. Wang, K. Zhang, H. Pan, W. Liu, Z. Zuo, *J. Am. Chem. Soc.* **2020**, *142*, 6216–6226.

[2] A. P. De Jong, S. W. Fesik, A. Makriyannis, *J. Med. Chem.* **1982**, *25*, 1438–1441.

[3] D. Orekhov, *Org. Process Res. Dev.* **2024**, *28*, 1032–1054.

[4] D. Chowdhury, R. Sutradhar, A. Paul, A. Mukherjee, *Chemistry A European J* **2024**, *30*, e202400942.

[5] D. M. Jackson, R. L. Ashley, C. B. Brownfield, D. R. Morrison, R. W. Morrison, *Synthetic Communications* **2015**, *45*, 2691–2700.

[6] A. J. Wilson, M. Masuda, R. P. Sijbesma, E. W. Meijer, *Angew Chem Int Ed* **2005**, *44*, 2275–2279.

[7] N. E. Behnke, R. Kielawa, D.-H. Kwon, D. H. Ess, L. Kürti, *Org. Lett.* **2018**, *20*, 8064–8068.

[8] S. Guizzetti, A. Michaut, G. Federspiel, J. Eymard, I. Caron, S. Quatrevaux, E. Daras, S. Jolly, J. Guillemont, D. Lançois, *Org. Process Res. Dev.* **2020**, *24*, 729–733.

[9] Y. Chen, D. Cantillo, C. O. Kappe, *Eur J Org Chem* **2019**, *2019*, 2163–2171.

[10] J. Yu, M. Lu, *Org. Biomol. Chem.* **2015**, *13*, 7397–7401.

[11] T. Betke, P. Rommelmann, K. Oike, Y. Asano, H. Gröger, *Angew Chem Int Ed* **2017**, *56*, 12361–12366.

[12] A. Yamamoto, K. Tanaka, Y. Hashimoto, N. Morita, O. Tamura, *Chemistry A European J* **2024**, *30*, e202303790.
